# Potential long-term, global effects of enhancing the domestic terrestrial carbon sink in the United States through no-till and cover cropping

**DOI:** 10.1186/s13021-024-00256-2

**Published:** 2024-06-14

**Authors:** Maridee Weber, Marshall Wise, Patrick Lamers, Yong Wang, Greg Avery, Kendalynn A. Morris, Jae Edmonds

**Affiliations:** 1 Joint Global Change Research Institute (Pacific Northwest National Laboratory and University of Maryland), University Research Court, College Park, MD USA; 2 National Renewable Energy Laboratory, Denver W Pkwy, Golden, CO 15013 USA

**Keywords:** No-till, Cover crops, Conservation agriculture, Soil carbon, Carbon dioxide removal, DayCent, GCAM

## Abstract

**Background:**

Achieving a net zero greenhouse gas United States (US) economy is likely to require both deep sectoral mitigation and additional carbon dioxide removals to offset hard-to-abate emissions. Enhancing the terrestrial carbon sink, through practices such as the adoption of no-till and cover cropping agricultural management, could provide a portion of these required offsets. Changing domestic agricultural practices to optimize carbon content, however, might reduce or shift US agricultural commodity outputs and exports, with potential implications on respective global markets and land use patterns. Here, we use an integrated energy-economy-land-climate model to comprehensively assess the global land, trade, and emissions impacts of an adoption of domestic no-till farming and cover cropping practices based on carbon pricing.

**Results:**

We find that the adoption of these practices varies depending on which aspects of terrestrial carbon are valued. Valuation of all terrestrial carbon resulted in afforestation at the expense of domestic agricultural production. In contrast, a policy valuing soil carbon in agricultural systems specifically indicates strong adoption of no-till and cover cropping for key crops.

**Conclusions:**

We conclude that under targeted terrestrial carbon incentives, adoption of no-till and cover cropping practices in the US could increase the terrestrial carbon sink with limited effects on crop availability for food and fodder markets. Future work should consider integrated assessment modeling of non-CO_2_ greenhouse gas impacts, above ground carbon storage changes, and capital and operating cost considerations.

**Supplementary Information:**

The online version contains supplementary material available at 10.1186/s13021-024-00256-2.

## Background

Executive Order 14008 has set the target of putting the United States (US) on a path towards a net-zero carbon economy by 2050 [1]. The economy-wide mid-century goal aligns with the Paris Agreement to limit global mean temperature change to 1.5 °C by 2100. Globally, land-based mitigation strategies including alternative management practices in agriculture, forestry, bioenergy, and other land-use (AFOLU) have the potential to sustainably contribute roughly 25% (10–12 GtCO_2_-eq yr^−1^) of the 50 GtCO_2_-eq yr^−1^ mitigation needed by 2050 to deliver on the 1.5 °C target [2]. The global physical and practical potential of enhanced soil organic carbon (SOC) sequestration as part of AFOLU has been estimated at 1.4–2.3 GtCO_2_-eq yr^−1^ [3], or 14–23% of the total AFOLU contribution. This means that natural climate solutions, such as AFOLU and enhanced SOC management, will likely play a key role in US net-zero strategy, in addition to direct reductions in emissions.

Agricultural soils are prime targets for enhanced SOC storage because they are already under active human management [4]. A variety of approaches are available to increase SOC content in these soils while maintaining cultivation; including cover cropping, addition of biochar, and reduced or no-tillage. As a suite these management practices are referred to as conservation agriculture. In an overview of natural climate solutions, Griscom et al. calculated the global carbon storage potential of conservation agriculture to be just under 0.5 GtCO_2_-eq yr^−1^ [5]. This estimate is intended to be conservative, taking into account concerns by Powlson et al. [6] that the magnitude of SOC sequestration potential from no-till cultivation in particular might have been overstated in other estimates. Despite high variation in SOC accumulation across climate, crop-system, and soil properties under different conservation agricultural practices, taken holistically, shifts to such management would have a positive effect on broader agricultural outcomes, potentially reducing the total amount of cropland required [5]. This is because conservation agriculture is known to have a suite of co-benefits ranging from increased yields to erosion reduction, increased nutrient retention (and thus reduced fertilizer demand), and increased soil–water retention [7–12]. Agronomists and soil scientists argue that shifting to sustainable agricultural systems such as conservation agriculture will be essential to feed the future population in perpetuity, with benefits to climate mitigation being a further service [13–15].

As a result, many states in the US have already established incentive programs to facilitate the adoption of conservation agricultural practices [11]. Currently, the US Department of Agriculture (USDA) Natural Resources Conservation Service (NRCS) offers several programs to help support climate smart agriculture, some of which include incentives to increase soil health through reduced or no-tillage and cover cropping. For example, the NRCS’s Conservation Stewardship Program offers annual payments to landowners for implementing and maintaining conservation practices, like cover cropping [16]. Similarly, the Environmental Quality Incentives Program offers assistance to solve various land issues by developing individual conservation plans and can provide financial aid to implement these practices [17]. Additionally, the NRCS has recently partnered with Farmers For Soil Health to launch an incentive program specifically for cover crops on corn and soybean farms [18]. On top of the soil health improvements, some of these programs highlight carbon sequestration as an additional benefit of no-till and cover cropping.

The need for atmospheric carbon removals in stringent climate change mitigation scenarios may lead to a greater expansion of conservation agricultural practices, especially if a carbon price is set. It is unclear whether such policies may result in resource competition, particularly for land, that would cascade into impacts on agricultural trade and the global climate system. A key question is thus whether the expansion of the conservation agricultural practices and potential shifts in land use impact US agricultural commodity outputs. Changes in US agricultural trade may result in increasing production in regions with fewer environmental regulations, lower regulatory oversight, enforcement, or climate targets, ultimately offsetting any net emission reductions through domestic conservation agriculture. Here, we quantitatively assess the potential impacts of a domestic expansion in two key conservation agricultural practices, no-till and cover cropping, in the context of unintentional and peripheral global land, trade, and emissions impacts using an integrated, global modeling framework.

Global, long-term carbon management pathways are often simulated with integrated assessment models (IAMs) because they take into consideration the land-energy-human-climate system. IAM projections are prominently reported by the Intergovernmental Panel on Climate Change (IPCC) due to their long-term, integrated, multisectoral dynamic coverage of global agriculture and energy. The Global Change Analysis Model (GCAM) is well-suited to consider the larger supply–demand dynamics of land resources and the integrated impacts of no-till and cover crop agriculture for energy, food, feed, and fiber across all sectors of the economy and in a global context. Previous assessments show the practical potential of no-till and cover cropping practices, as well as some of the associated uncertainty. However, these assessments were not performed in an integrated economic context that considers the costs and trade-offs of changing crop production and alternative uses of land in a global market [2, 5].

Here, we analyze the potential scale and impact of expanded no-till and cover cropping agricultural practices in the US as part of an integrated land-use response to a carbon storage incentive. Our approach is to combine biogeochemical modeling of no-till and cover cropping practices for corn, fiber-crops (i.e., cotton), soybeans, wheat, and other grains (i.e., sorghum) with GCAM modeling of the decisions about economic and physical trade-offs among these practices. The Daily Century (DayCent) model integrates climate, soil, crop-type, and field management information, and includes linked carbon, nutrient, and water biogeochemical cycles. It thus can simulate the effects of different land uses or field management strategies under varied environmental conditions. Within GCAM, we used exploratory scenarios to determine the sensitivity of the model to valuing terrestrial carbon (Table 1). We then address the integrated-assessment impacts of no-till and cover cropping in the US in comparison to a reference scenario with no carbon price.

**Table 1:** Scenario names and descriptions

Scenario/Sensitivity	Description
All Carbon (AC), sensitivity	A US-based carbon policy that values all terrestrial carbon, regardless of sector
Cropland Carbon (CC), sensitivity	A US-based carbon policy that values specifically soil carbon in agricultural systems
Reference with Protected Land (REF)	A reference scenario where no-till and cover crop agricultural practices are implemented into GCAM in the US. 90% of previously undeveloped lands are protected from expansion of managed land-use in the US, with no fiscal incentive for carbon storage
Cropland Carbon with Protected Land (CCPL)	A US-based carbon policy that values soil carbon in agricultural systems in the US, with 90% of previously undeveloped lands protected from expansion of managed land-use in the US

## Methods

The “Biogeochemical model calibration and validation” section of Methods includes information about our meta-analyses of literature on no-till and cover cropping, and how DayCent was used to parameterize GCAM. The “The Global Change Analysis Model (version 6.0)” section provides an overview of GCAM, with “Integrated assessment modeling of no-till and cover crop agricultural practices” detailing how no-till and cover crop agricultural practices were modeled in GCAM, and “Carbon price pathway” explaining our carbon price pathway.

## Biogeochemical model calibration and validation

Both cover cropping and no-till have been extensively researched as soil carbon management strategies and for their effect on crop yields. A summary of meta-analyses examining the effects that cover crops have on primary crop yield and SOC is shown in Additional file 1: Table S1. Several meta-analyses state that the addition of cover crops will increase SOC, as a weighted mean effect across all climate types, primary crops, and land management options evaluated globally [6, 19, 20]. Yield can decrease slightly when either legume or non-legume cover crops are planted, although a mix of these cover crop types can increase yield (on average for primary crops) [21]. For maize specifically, there was an increase for both legume and mixed cover crops in the U.S and Canada [22], and in arid climates yield decreases with cover crops under all circumstances due to increased competition for water [23]. The largest source of variation in cover crop impacts derives from the type of cover crop that is planted. Legume cover crops have the ability to fix nitrogen in the soil through their root endosymbionts, which can lead to greater nitrogen availability compared to the same growth of primary crops with either no cover crops or non-leguminous covers. For this reason, we chose to model three cover crop options: legume, non-legume, and fallow (none).

The impact of no-till management on SOC is variable. This is partially driven by tillage redistributing SOC within the soil profile, which can result in loss of SOC at depth when tillage ends [20]. Therefore, in some instances total SOC stock does not change after the adoption of no-till. The current understanding of this variability is well reviewed in both Powlson et al. (2014) and Ogle et al. (2019) [6, 20]. In roughly half of all studies, there is an increase in SOC after converting to no-till, and this is reflected in our model calibration. A recent review [10] has pointed out that there was room for improvement in the comparison of no-till vs. conventional tillage based on the selection criteria for trials used in the meta-analysis. Nicoloso et al. [10] shows that it was mainly high-inversion tillage compared to no-till that showed carbon loss at moderate soil depths. For all other tillage methods, no-till had positive or no change in soil carbon at all depths, with more carbon sequestered in the soil overall. Several reviews have the similar conclusion that no-till can decrease crop yield on average [24–26], with this effect more pronounced in humid climates- yield differences between no-till and conventional till are either negligible or much smaller in dry regions. This finding is an overall result, with the widest yield changes exhibited in the first one to two years after adopting no-till [25], and differences in yield are reduced when residue is retained on the field [24].

For both cover cropping and no-till, existing literature values (Additional file 1: Table S1) were used to compare if the results obtained from DayCent calibration align with expected trends. Values input to DayCent were taken from individual field trials that incorporated a cover crop, primary crop, and geographic location. Generalized literature results were only used as a point of comparison after DayCent runs were completed.

To evaluate long-term effects of no-till and cover crops on primary crop yield and SOC, the DayCent biogeochemical model [27, 28] was adopted to simulate carbon and nitrogen dynamics under all GCAM technology treatments in representative water basin-crop systems. To focus our study on crops that capture the greatest carbon mitigation potential, the five field crops with the largest number of harvested acres according to the 2017 National Agricultural Statistics Service (NASS) Census of Agriculture were selected for simulation [29]. Spatially, GCAM conducts simulations at the water basin level. Thus, water basins with over five million hectare (Mha) growth of selected crops were then chosen to represent major cropping system regions. The selection procedures determined 30 water basin-crop combinations, covering 75% of the total US cropland and over 90% of the no-till relevant US cropland.

**Fig. 1:**
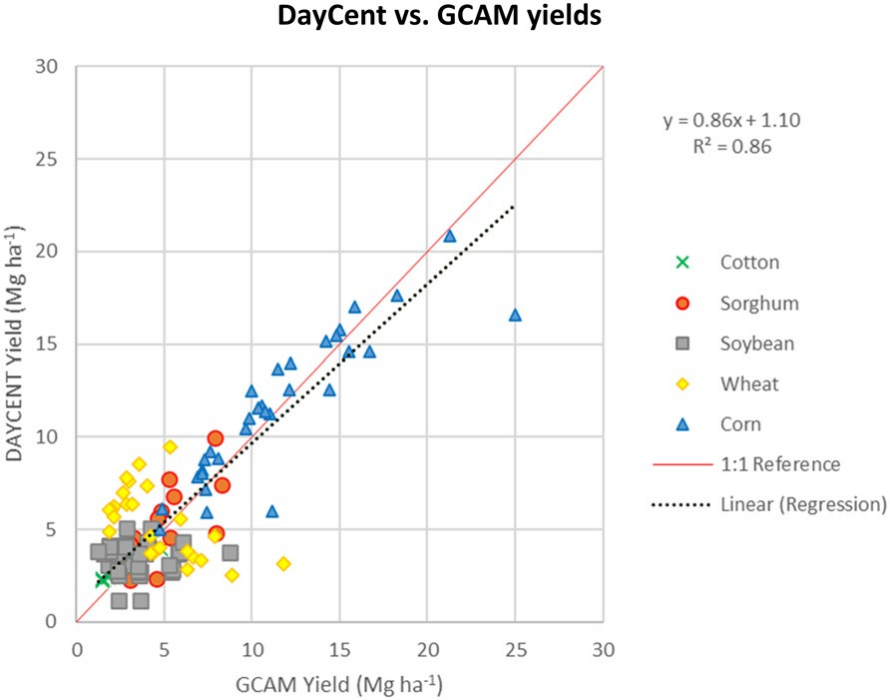
Verification of DayCent performance by fitting yields from DayCent output to GCAM yield database. The US winter wheat growing areas (according to NASS) have very distinct and heterogeneous conditions. This explains the deviation from the mean

**Fig. 2.**
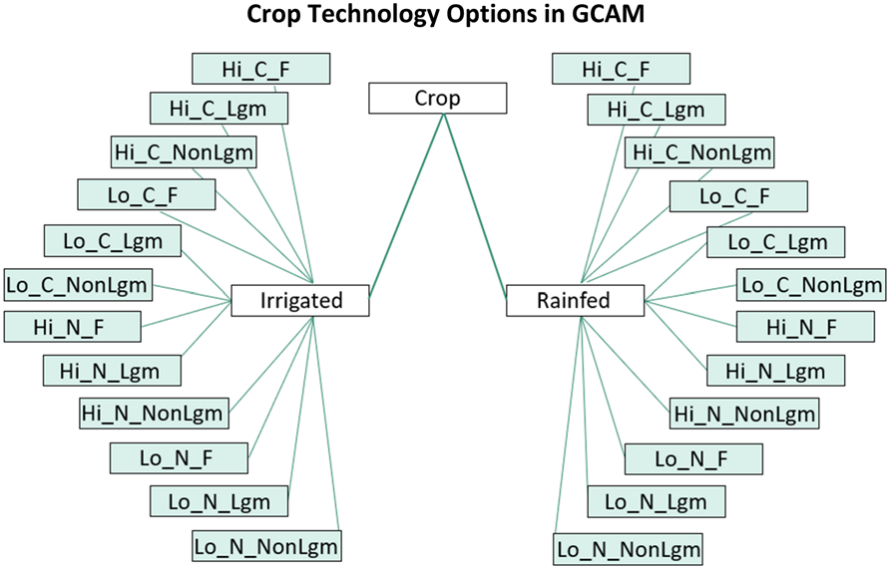
24 technology options within a given crop include irrigated or rainfed, high (Hi) or low (Lo) fertilizer application rates, conventional tillage (C) or no-till (N), and legume (Lgm), non-legume (NonLgm), or fallow (F) cover cropping

The DayCent projections were conducted for the period of 2016–2100, based on 5,000 years of spin-up runs ending in 1850, and 215 years of baseline runs from 1851–2015. Calibration and validation of DayCent was conducted by fitting yields of the most recent years prior to projections (i.e., circa 2015) from DayCent to those from GCAM under different irrigation and fertilization technologies (Fig. 1). The projections used Daymet weather data [30] from 1986–2015 and circulated this input to the end of the century without considering potential climate changes. Since we are focusing on major crops on productive cropland, soil was characterized as a typical loamy texture for all simulations, with 40% sand, 40% silt, and 20% clay. Each of the 30 water basin-crop combinations was associated with 24 technology treatment combinations composed of: irrigated and rainfed water management, high and low fertilization rates, conventional tillage and no-till, and legume cover crops, non-legume cover crops, or no cover crop (fallow) (Fig. 2). Thus, a total of 720 combination runs were conducted in DayCent to parameterize yields and SOC in GCAM.

## The Global Change Analysis Model (version 6.0)

**Fig. 3:**
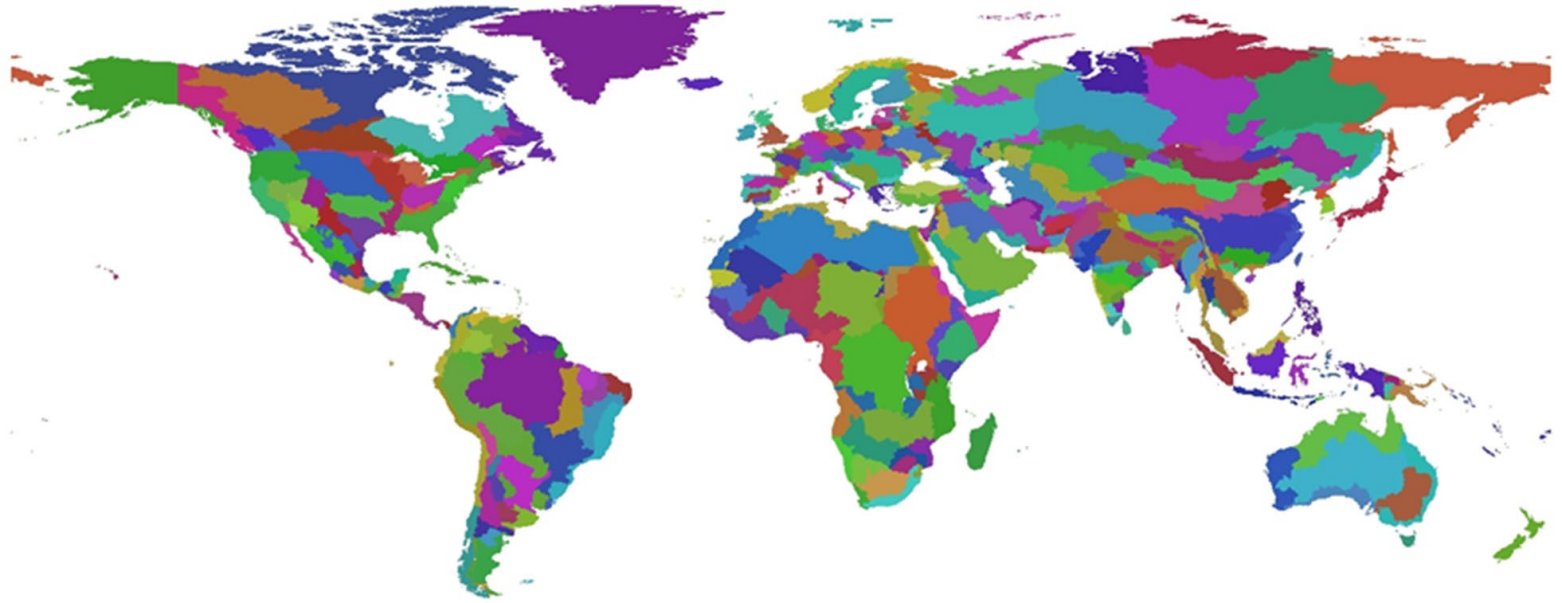
Land use regions in the GCAM model (based on the intersection of 32 energy-economy regions and 235 global water basins)

GCAM is a well-established, long-term, multisector integrated, human and earth systems model that links a global energy-economy-agriculture-land-use model with a climate model of intermediate complexity [31, 32]. GCAM models the global energy system with a spatial resolution of 32 regions, and agriculture and land use with a spatial resolution of 384 regions based on the intersection of energy regions and water basins (Fig. 3). The analysis in this study was developed using GCAM version 6.0. GCAM and its predecessors have long been prominent in analyzing the impact of policies on bioenergy, agriculture, and land use emissions [33, 34]. Global population, a key driver of food demand growth, is assumed to peak at 9.5 billion by 2070 before declining slightly to the end of the century (see Calvin et al. 2019 [37] for a detailed description and sourcing of socio-economic drivers and other model details). Agriculture and land-use modeling are based on economic equilibrium with physical land units preserved, with all commercial and natural lands and their terrestrial carbon stores represented [35]. GCAM has been used for a variety of agriculture and land use related studies, including some that address land use change emissions and international agricultural trade [35–39]. GCAM is an open-source, publicly available, community model that can be downloaded at http://jgcri.github.io/gcam-doc/index.html.

### Integrated assessment modeling of no-till and cover crop agricultural practices

For this study, we added no-till and cover crop options to the most relevant crops and crop management practices in the US contained in GCAM 6.0. With the inclusion of new management practices, the existing agricultural technologies (baseline) in GCAM were considered by default to reflect conventional tillage without cover crops. However, it should be noted that baseline GCAM yield and SOC values implicitly include no-till and cover crop practices, as the yields, fertilizer application, water demands, and soil carbon associated with existing technologies reflect all agriculture production. In other words, until this current assessment, conventional and conservation (no-till, cover crop) management practices were not distinguished in GCAM, so values in the base year (2015) and previous years should not be interpreted as representing conventional technologies alone.

Because GCAM considers crop yield variation in terms of percent change, while DayCent deals in yield directly, GCAM values were harmonized so the baseline inputs for conventional tillage technologies were comparable. For each relevant water basin and agricultural technology in GCAM, a yield index was created by setting the yield in 2015 to a value of 1.0, and using yield change to calculate future yield. The DayCent output was used to calculate average yield ratios, with the ratio being the yield for a conventional-fallow technology to its no-till/cover crop counterpart. The yield change values in gcamdata [40] are provided in 5-year intervals while DayCent output was provided annually, so the yield ratios from DayCent were averaged to match the periods used in GCAM. Then, for each technology, the yield ratio from DayCent was used to capture the change from conventional-fallow to no-till/cover crop, and this change was added to the yield index from gcamdata. This resulted in values that reflected both the yield changes in GCAM due to improved technology, as well as those from converting from conventional to no-till and cover crop agricultural practices. Finally, the modified yield index was used to calculate new yield change values that represent the new technologies.

For SOC, a similar approach was taken. SOC values that already existed in gcamdata were modified, and additional adjustments were made to the temporal dynamics of SOC accumulation. In GCAM, every agricultural technology is assigned a soil carbon density value that represents the peak amount of carbon that can be stored by that specific regimen. For the US, under default parameters, maximum carbon density for a specific land-use by region combination is reached after 50 years of consistent management. Using DayCent output, this equilibrium point was adjusted to reflect varying SOC accumulation with different management practices. Annual SOC values for each technology were smoothed using a cubic polynomial. Then, the equilibrium point was identified as either: 1) the year that SOC reached its maximum value or 2) the point at which the rate of change was closest to zero without being negative. The years needed to reach SOC equilibrium were calculated as the DayCent model year at the equilibrium point minus 2015 (the year of onset for DayCent runs). This resulted in each technology having a year in which SOC equilibrium is met and a value for the SOC at equilibrium from DayCent. The conventional technology SOC at equilibrium from DayCent was then scaled to match the corresponding value in GCAM, and the no-till and cover crop agricultural technologies were scaled by that same factor. This adjustment is shown below.

$${\text{Scaling Factor }} = {\text{ DayCent}}\left( {{\text{SOC}}_{{\text{max}}} } \right) \, /{\text{ GCAM}}\left( {{\text{SOC}}_{{\text{max}}} } \right)$$

where DayCent(SOC_max_) is from conventional agricultural practices, and GCAM(SOC_max_) is the default model value for each water basin by crop combination. For all other technologies:

$${\text{GCAM}}\left( {{\text{SOC}}_{{\text{max}}} } \right) \, = {\text{ DayCent}}\left( {{\text{SOC}}_{{\text{max}}} } \right){\text{ x Scaling Factor}}$$

**Fig. 4:**
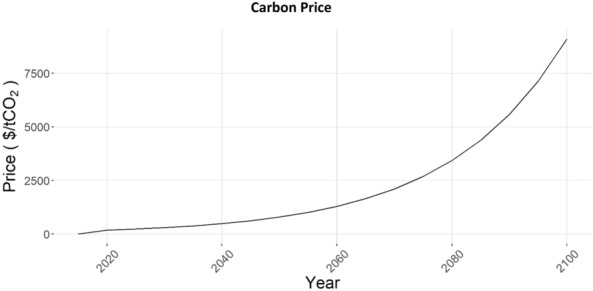
Carbon price pathway used for AC and CC sensitivities, and CCPL scenario

After the yield and SOC adjustments were made, shares of each technology were set in 2020 based on a report from the USDA Economic Research Service (ERS) [11]. To assess the impacts of increased no-till and cover crop practices and their potential as terrestrial carbon storage options in GCAM, a cropland-carbon policy scenario was generated. The details of this scenario were informed by sensitivity analysis of GCAM to carbon pricing in different land-use contexts (Table 1). For scenarios with carbon valuation, the carbon price pathway shown in Fig. 4 was used.

### Carbon price pathway

The carbon price pathway was chosen to be reflective of a deep global mitigation scenario where carbon emissions and carbon uptake in the energy and land use systems is either penalized or incentivized, respectively, with the actual prices given less importance compared to the direction of carbon flow. The price trajectory shown in Fig. 4 is based on GCAM scenarios constructed to reflect a realistic pathway. The starting value in 2020 is $183.50, based on a recent study that estimated the current social cost of carbon [41]. This value increases at a modest rate of 5% annually through 2100. Additional carbon price pathways were tested with key metrics analyzed across the pathways, shown in Additional file 1: Figures S1 and S2. For this study, the carbon price trajectory is just imposed on the US in the scenario and sensitivities in which it is applied. For the All Carbon (AC) sensitivity, an annuity based on the carbon price is assumed paid for land uses that increase and hold terrestrial carbon, in both vegetation and soil. This incentive is applied not just to cropland, but to all land uses including forests, which is a large driver of the results. For both the Cropland Carbon (CC) sensitivity and the Cropland Carbon with Protected Land (CCPL) scenario, this annuity for expanding and holding carbon stocks is applied exclusively to soil carbon in cropland. By applying a carbon price to only land use in cropland, we mimic the response of having a subsidy on the cropland that would encourage cropland owners to switch to using no-till and/or cover crops.

## Results

The “Land allocation” section of Results covers impacts on agricultural land allocation, with “Land allocation for individual crops” focusing on individual crops and their yield and SOC dynamics. The “Crop production” section presents results for changes in crop production, with “Crop prices and trade” covering the related crop price and trade implications. Finally, terrestrial carbon and emissions results are shown in the “Terrestrial carbon” section.

## Land allocation

Adoption of no-till and cover cropping and allocation of land to plant-based agriculture varied considerably among the two scenarios and sensitivities. Land allocation results from the AC and CC sensitivity scenarios helped inform the development of the Reference with Protected Land (REF) and CCPL scenarios. The AC and CC scenarios saw strong afforestation and deforestation responses, respectively, and details can be found in the supplemental (Additional file 1: Appendix I).

**Fig. 5:**
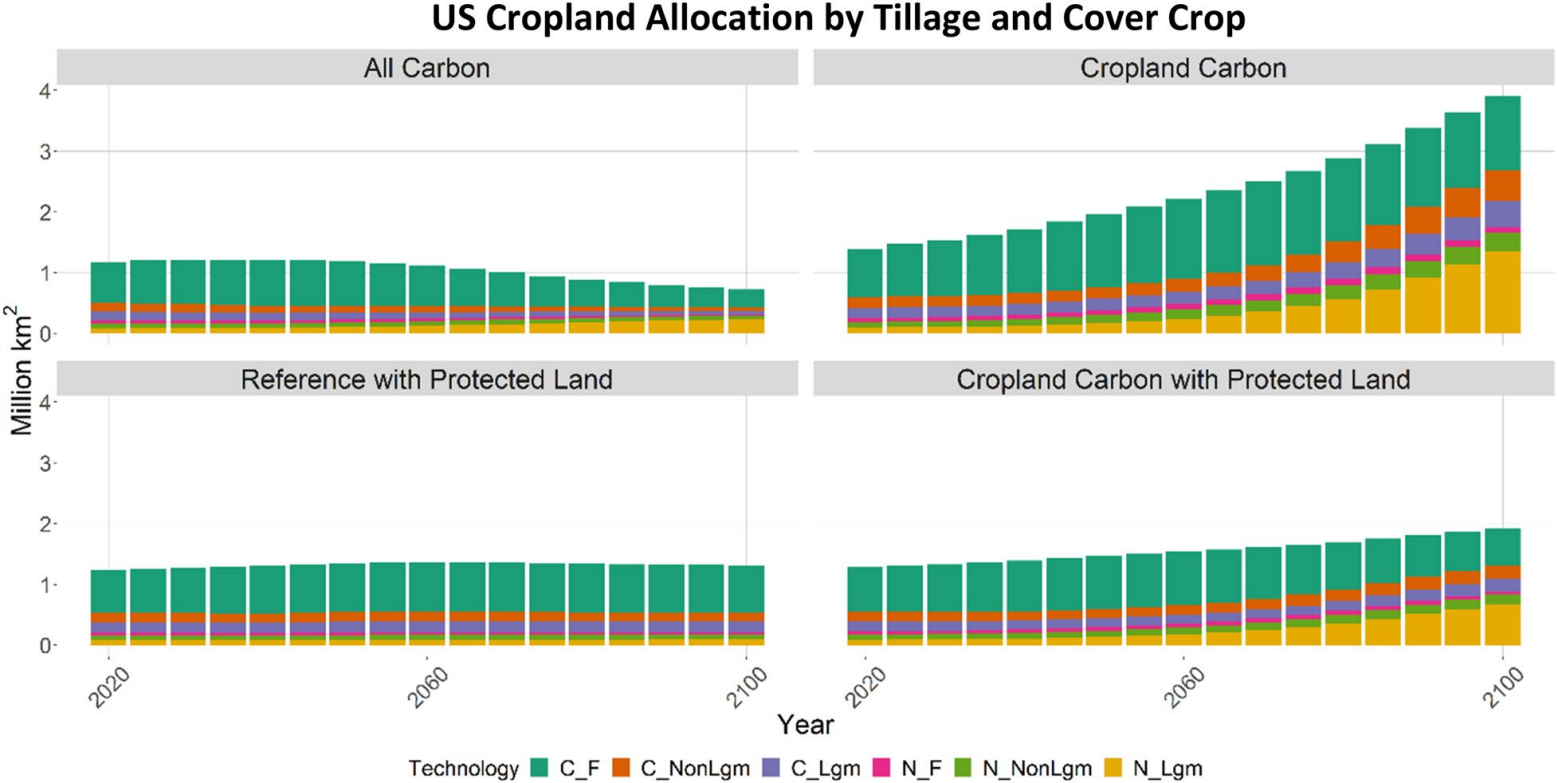
Total cropland allocated to aggregate agriculture technologies in the US, by scenario and sensitivity. Management regimes: C: conventional tillage; N: no-till; F: Fallow; Non-Lgm: Non-legume cover crop; Lgm: Legume cover crop

In REF, which reflects integrated responses to new technology options without any explicit carbon storage incentive, total cropland in the US remains fairly constant through the end of the century (Fig. 5). Land managed using a no-till legume (N_Lgm) regimen increases slightly by the end of the century, but a majority of the expansion is in conventional-fallow (C_F) technology. This result can be attributed to the absence of a carbon valuation which results in less direct incentive to switch to no-till and cover cropping beyond yield increases. The results of the CCPL scenario see cropland expanding moderately compared to REF by 2100, and no-till and/or cover cropping practices gradually taking over majority of the total allocation. By 2100, conservation agricultural technologies represent 68% of total cropland in the US, with almost two-thirds of that land combining no-till with cover crops. This scenario resulted in less of a forest response globally compared to the AC and CC sensitivities (see Additional file 1: Figures S3, S4, and S5 in Appendix I).

### Land allocation for individual crops

**Fig. 6:**
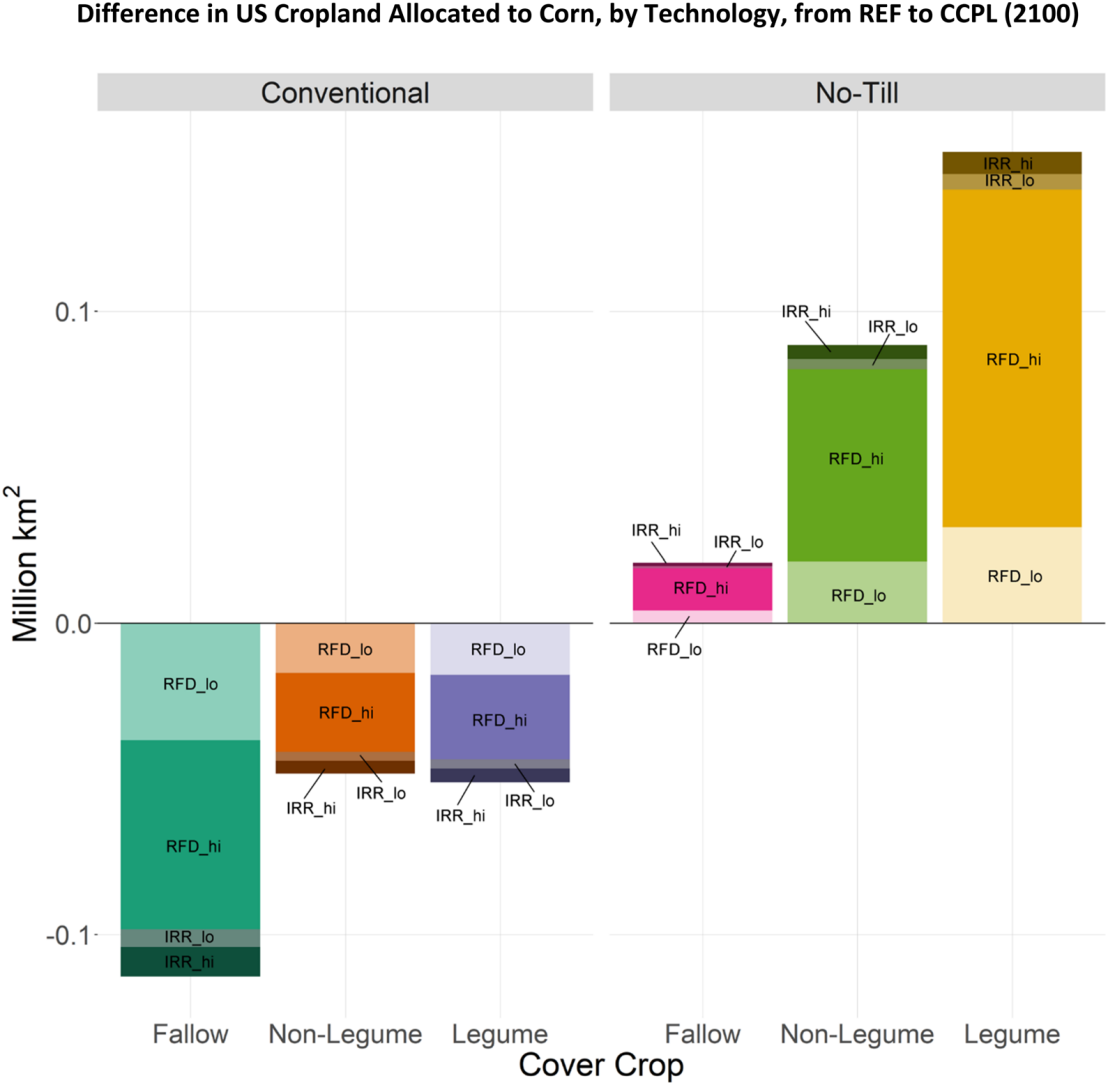
Difference in total cropland allocated to corn technologies in the US (CCPL – REF) in 2100. Additional technology options: IRR: Irrigated farmland; RFD: Rainfed farmland; hi: High fertilization rate; lo: Low fertilization rate

**Fig. 7:**
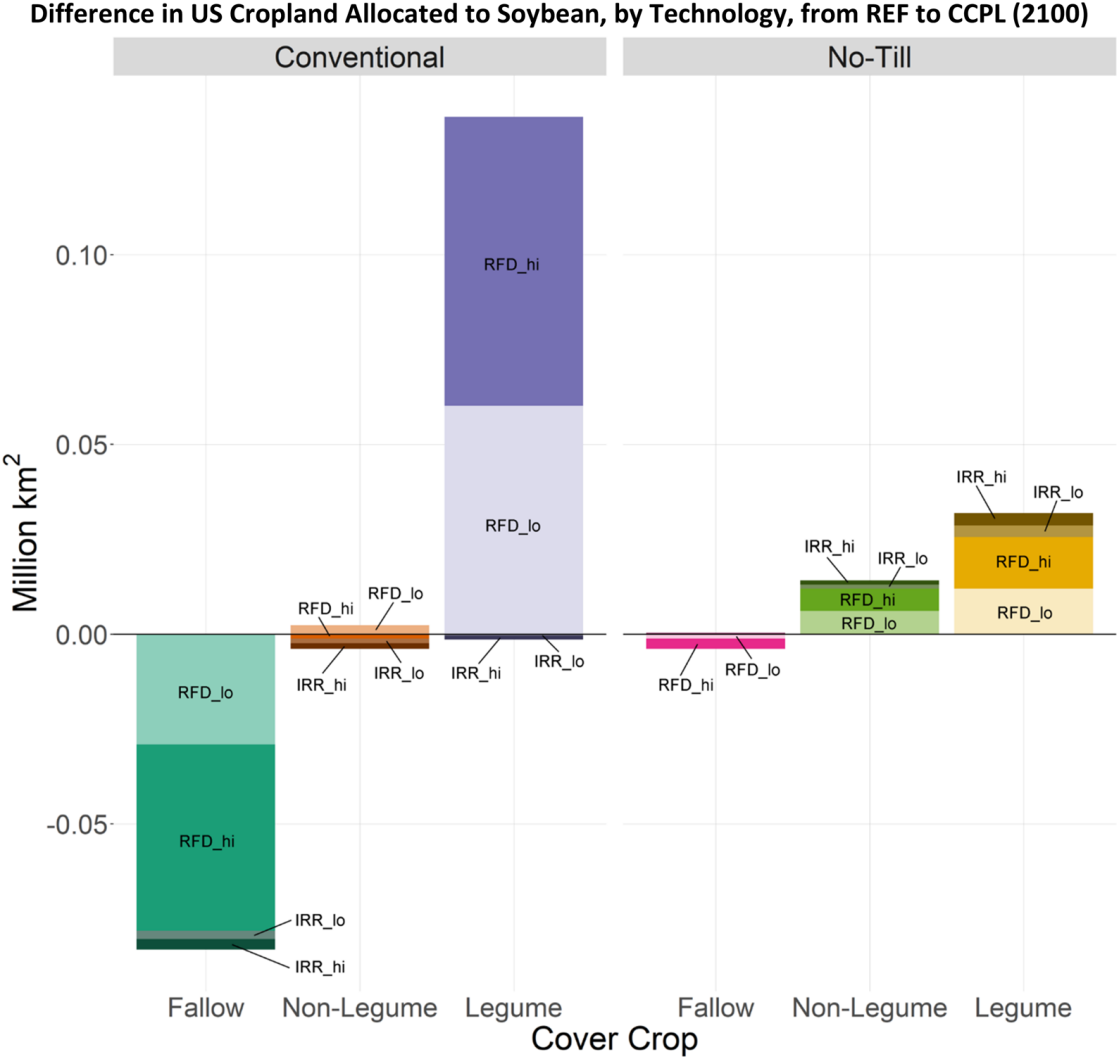
Difference in total cropland allocated to soybean technologies in the US (CCPL – REF) in 2100. Difference in cropland allocated to no-till irrigated fallow technologies were negligible. Additional technology options: IRR: Irrigated farmland; RFD: Rainfed farmland; hi: High fertilization rate; lo: Low fertilization rate

**Fig. 8:**
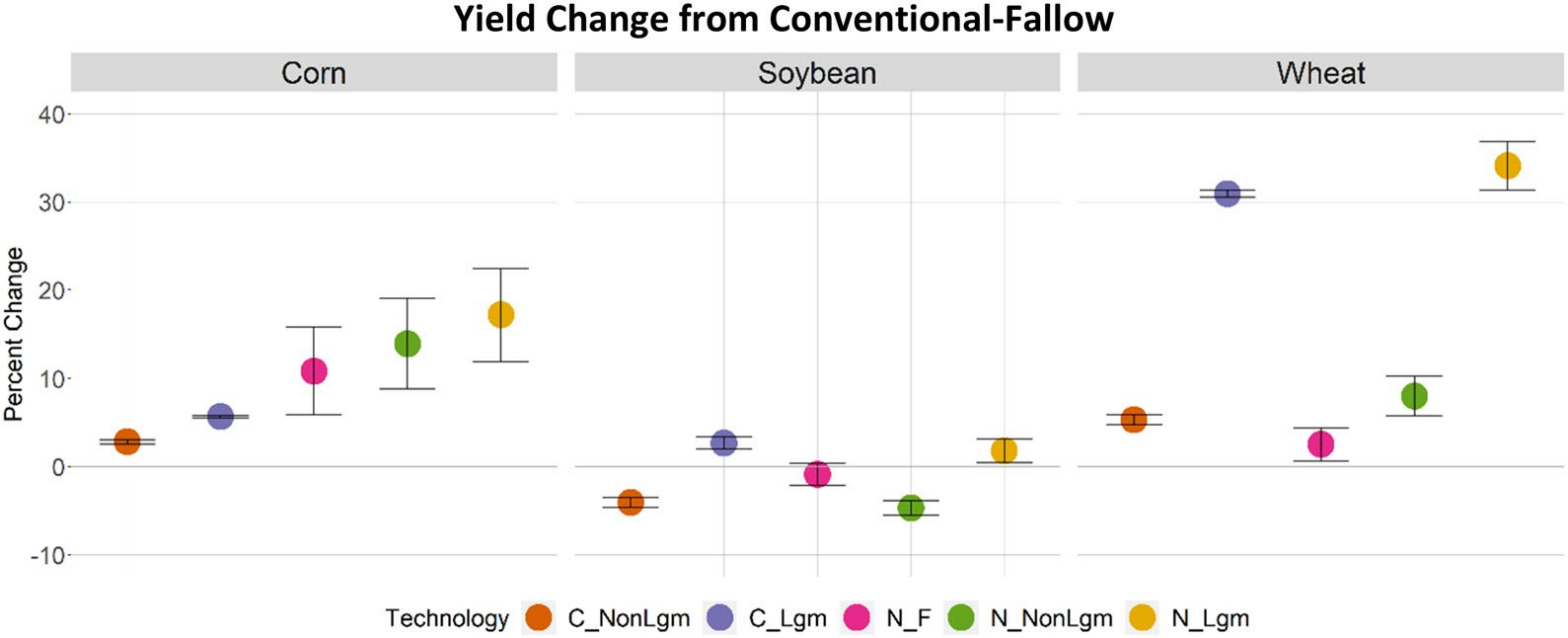
Percent change in yield from conventional-fallow technology for corn, soybean, and wheat. Error bars represent one standard deviation of the mean of irrigated/rainfed and hi/lo technologies, averages are plotted

**Fig. 9:**
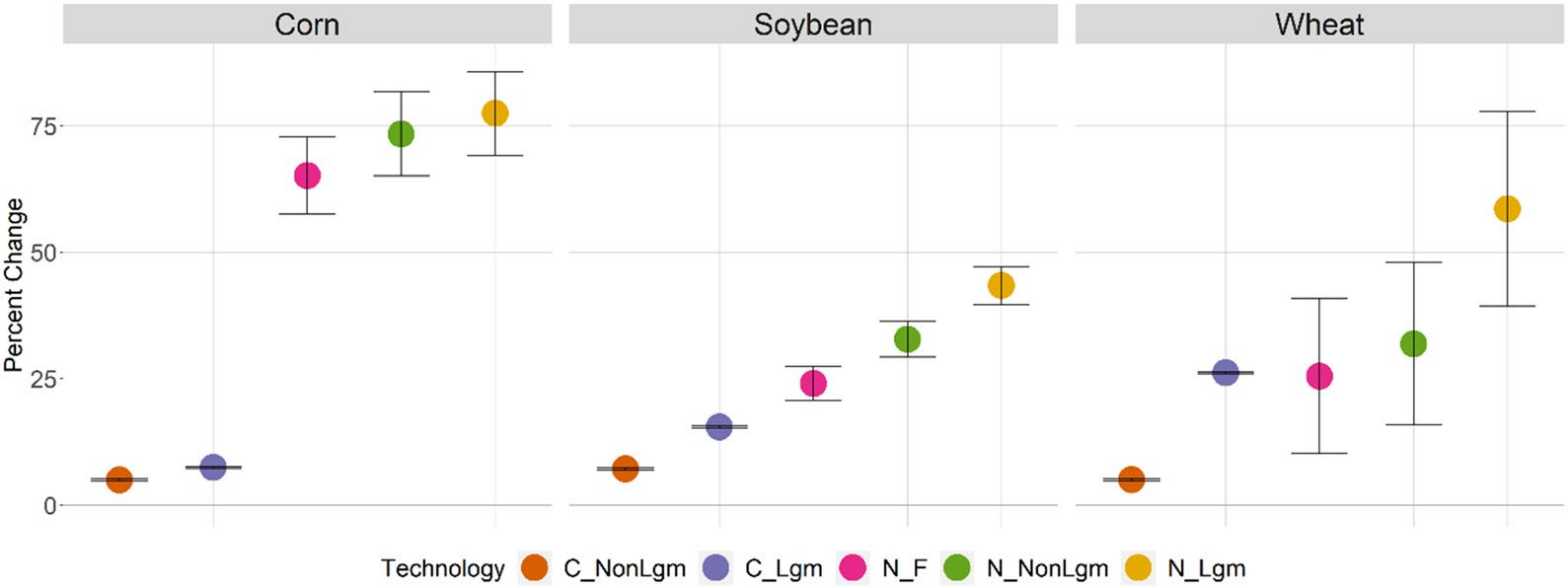
Percent change in SOC potential from conventional-fallow technology for corn, soybean, and wheat. Error bars represent one standard deviation of the mean of irrigated/rainfed and hi/lo technologies, averages are plotted

The no-till and cover crop agricultural practices are modeled at the level of individual crops in the US, where the physical impact of the practice on yields and SOC will differ, with corresponding differential impacts on land allocation and other results. For corn, by 2100 we see an increase in all no-till technologies in CCPL from REF (Fig. 6), with most of the area being rainfed and utilizing cover crops (Legume and Non-Legume). Alternatively, for soybeans, while the share of cropland managed as no-till with cover crops increases in CCPL, conventionally-tilled legume management also increases (Fig. 7). The differing technology behaviors between these two crops is attributable to differences in yield and SOC storage potential (Figs. 8 and 9). No-till corn offers higher yields and much greater SOC storage potential than its conventional tillage equivalent, and while these benefits are also present under soybean, they are not as substantial (from results found via DayCent calibration and modeling from present day through 2100).

There were marked differences in average yield and SOC for corn, soybean, and wheat when comparing no-till and cover cropping to conventional-fallow management (Figs. 8 and 9). These changes can explain the land allocation behavior shown in Figs. 6 and 7, and are based on DayCent output rather than a specific GCAM scenario. These combined results show that all technology options for corn provide yield improvements when switching from conventional-fallow crop management (Fig. 8), with no-till having a stronger impact than cover crops when adopted in isolation. However, greater yield improvements can be achieved through combining no-till and cover crop practices. Conversely, for soybean, we see minor yield improvements when a legume cover crop is used, regardless of tillage. In management practices without a cover crop or with a non-legume cover crop, yields were lower for soybeans than under conventional-fallow, again regardless of tillage. DayCent output shows drastic increases in SOC potential for corn when switching from conventional tillage to no-till, while for soybeans this increase is moderate (Fig. 9). This, in combination with the yield change, explains why no-till and cover crop agricultural technologies only mildly expand on land allocated to soybeans, while cropland allocated to corn becomes dominated by them.

**Fig. 10:**
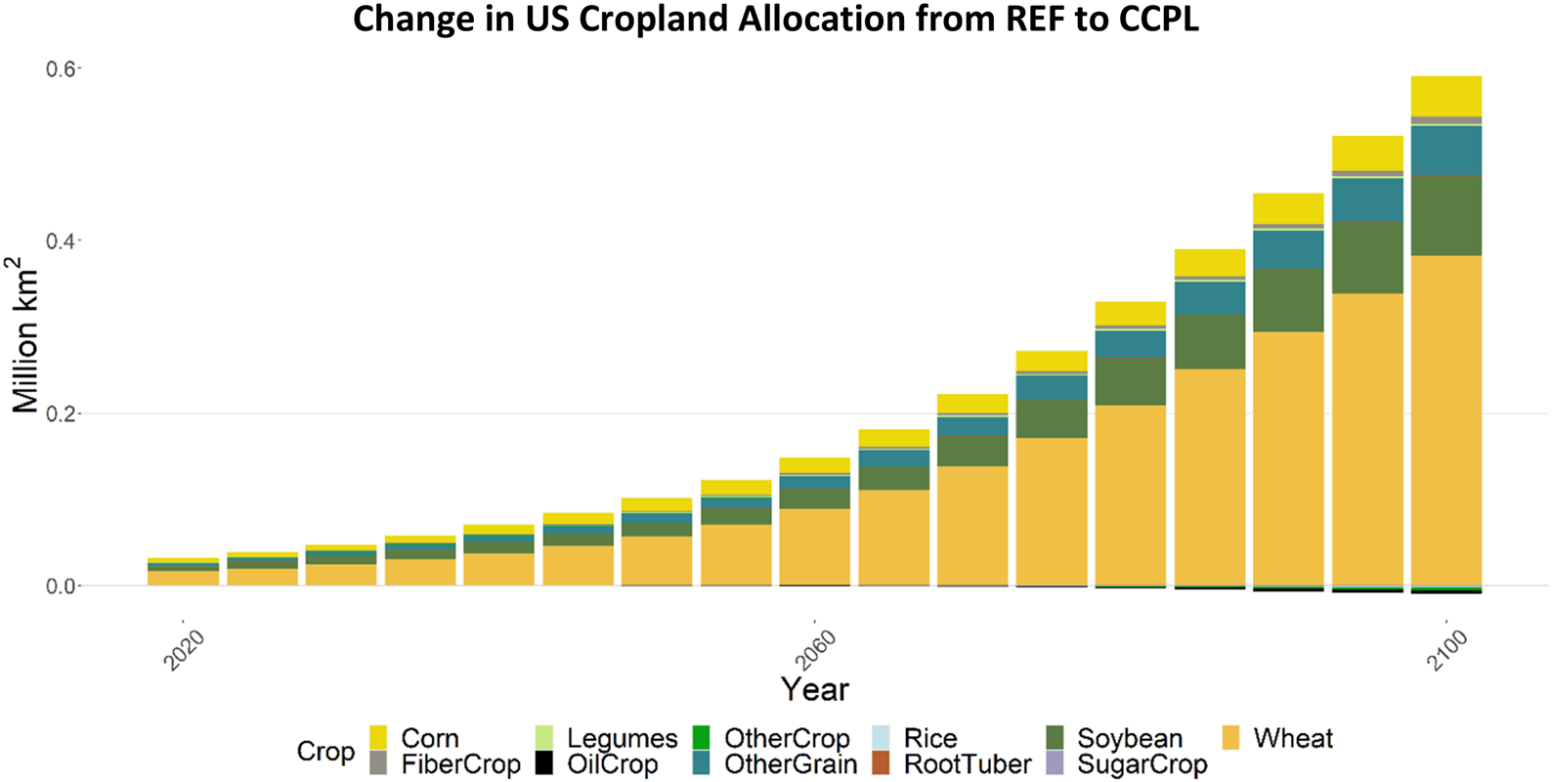
Change in cropland allocation, by crop, in the US (CCPL – REF). “OtherCrop” is an aggregate group of fruits, nuts, seeds, vegetables, purpose-grown biomass, and fodder

**Fig. 11:**
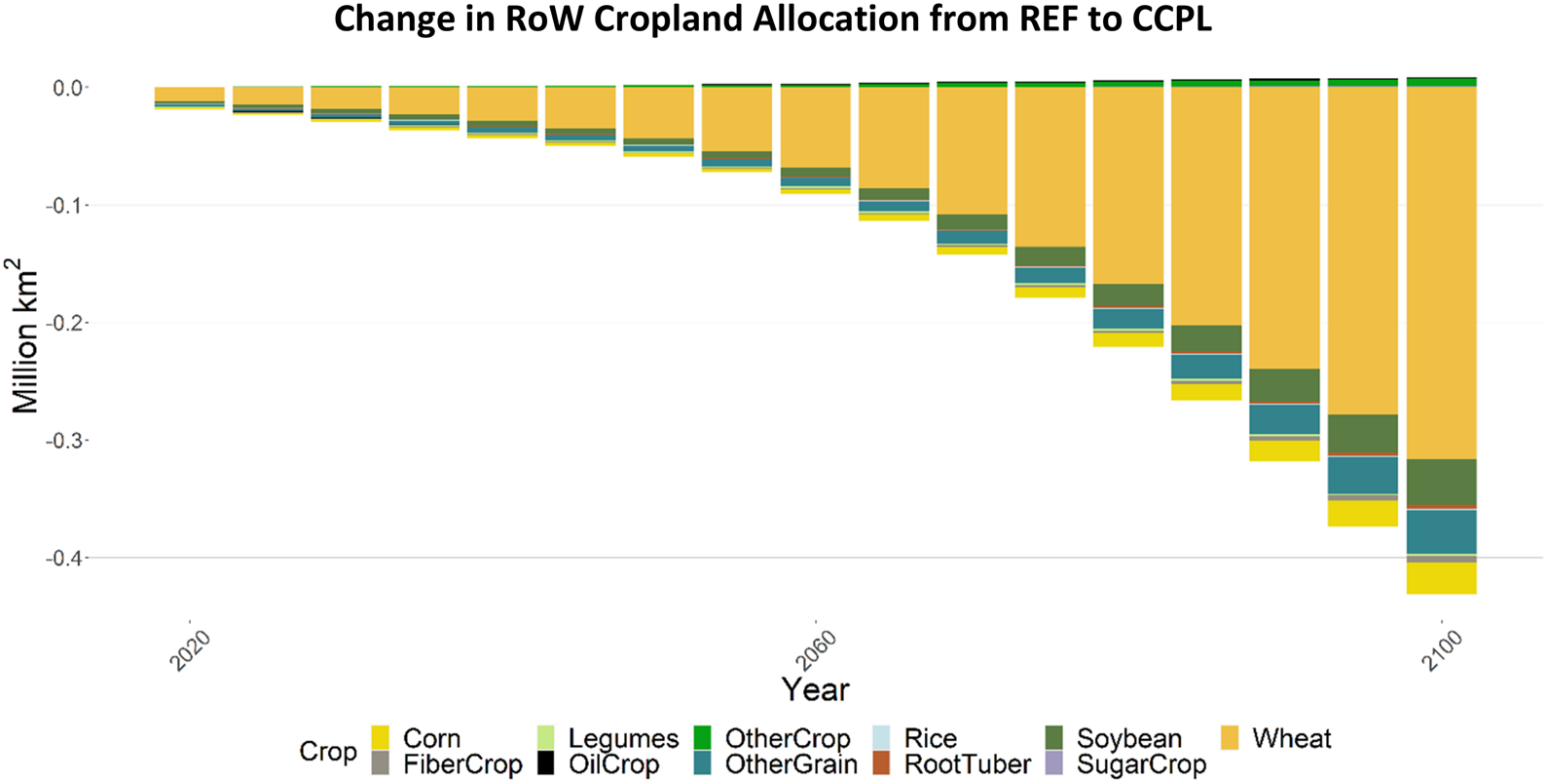
Change in cropland allocation, by crop, in RoW (CCPL – REF). “OtherCrop” is an aggregate group of fruits, nuts, seeds, vegetables, purpose-grown biomass, and fodder

Additionally, comparing the yield and SOC differences between soybean and wheat explains differences in land allocation to these crops both within the US and globally (Figs. 10 and 11). We found that land allocated to (and production of) wheat increases significantly in the US in the CCPL scenario compared to the REF scenario, while both metrics increase much less for soybean (Fig. 10). SOC storage for soybean and wheat do not vary as strongly between conventional and no-till technologies compared to corn (although wheat can achieve greater SOC improvements, Fig. 9). The major difference between these crops is their change in yield with adoption of no-till and/or cover cropping (Fig. 8), with wheat showing much greater yield improvement when planted with legume cover crops. This drives an increase in US cropland allocation to wheat and decreases it in other countries. Yield and SOC changes for fiber crop and other grain are shown in Additional file 1: Figures S6 and S7, respectively.

Figures 10 and 11 compare impacts on cropland allocation in the US (Fig. 10), and in all other regions excluding the US (Rest of World [RoW], Fig. 11). In the US, land allocated to wheat increases the most of all crops by the end of the century in CCPL (Fig. 10). Relative to its total allocation in the US in REF (Additional file 1: Figure S8), this increase in wheat allocation gives it the largest single share of cropland in the US by 2100 in CCPL, though almost 50% of cropland is still dedicated to corn and soybean. Land allocated to soybean, other grain products (e.g., barley, rye, quinoa), other fiber crops (e.g., cotton), and corn in the US also increases in CCPL, but by much less. While it may be unexpected that land allocated to corn only increases slightly, despite having improved yields and increased SOC storage, this change must be viewed in comparison to the other crops in this study. Land allocated to wheat, for example, is dominated by a no-till legume technology by 2100, which offers an average 33% yield increase in addition to up to an 80% increase in SOC. Similar attributes apply to other grain, though the yield improvement is slightly lower than for wheat (Additional file 1: Figure S6). Total cropland in the US increases by around 0.6 million km^2^, and decreases in RoW by over 0.4 million km^2^ by 2100 (Fig. 11). This is a comparable tradeoff, with RoW reducing land allocated to wheat the most, as there is incentive for the US to grow more of this.

## Crop production

**Fig. 12:**
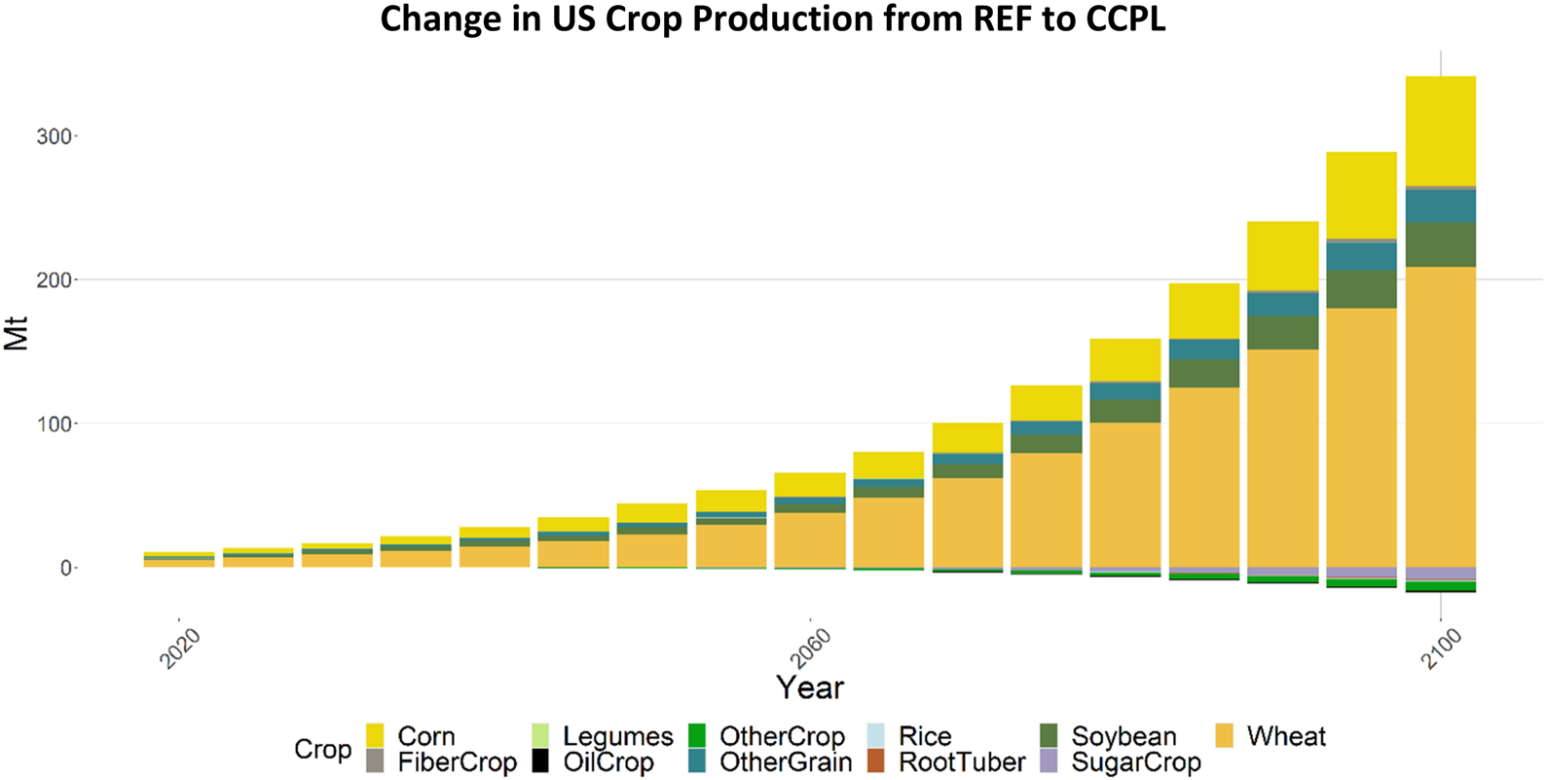
Change in crop production, by crop, in the US (CCPL – REF). “OtherCrop” is an aggregate group of fruits, nuts, seeds, vegetables, purpose-grown biomass, and fodder

**Fig. 13:**
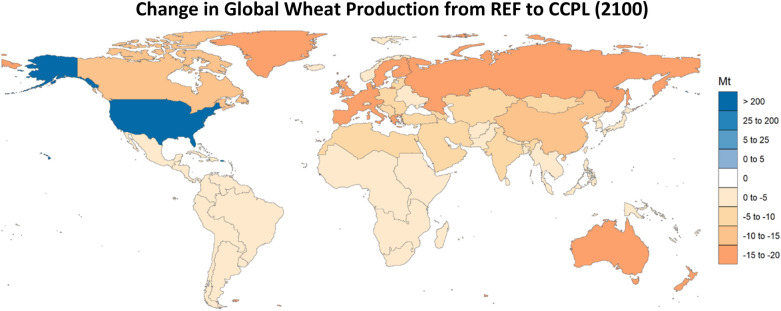
Change in wheat production by region in 2100 (CCPL – REF)

Due to yield improvements and changes in land allocation for crops in the US that have new no-till and cover crop agricultural technology options, we can expect to see changes in domestic production (Fig. 12). The four crops (corn, wheat, other grain, and fiber crop) that have higher yields across the board when switching from conventional practices to no-till and cover cropping are produced in greater quantities. Additionally, the production of soybean increases even though it has reduced yields for some no-till and cover cropping technologies. This increase in soybean production is the result of the use of conventional-legume practices, which have higher yields and SOC potential, comprising the largest share of land allocated to soybean. Globally, the CCPL scenario resulted in the strongest shifts in production for wheat, with a pronounced increase in wheat production in the US (208 Mt in 2100) mostly offset by decreases in production in all other regions (Fig. 13, total of 145 Mt in 2100), but still resulting in a net increase in global wheat production.

## Crop prices and trade

**Fig. 14:**
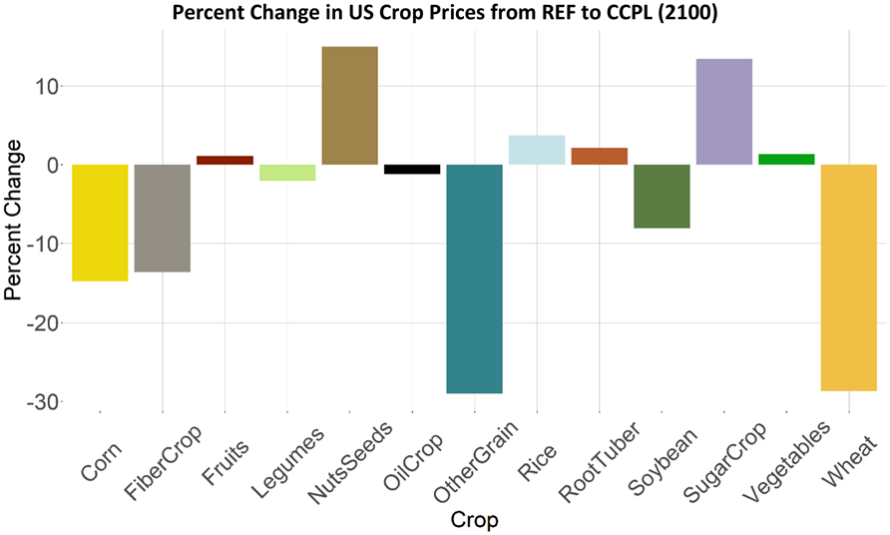
Percent change in US crop prices in 2100 from REF to CCPL. Negative values represent a decrease in price in CCPL

**Fig. 15:**
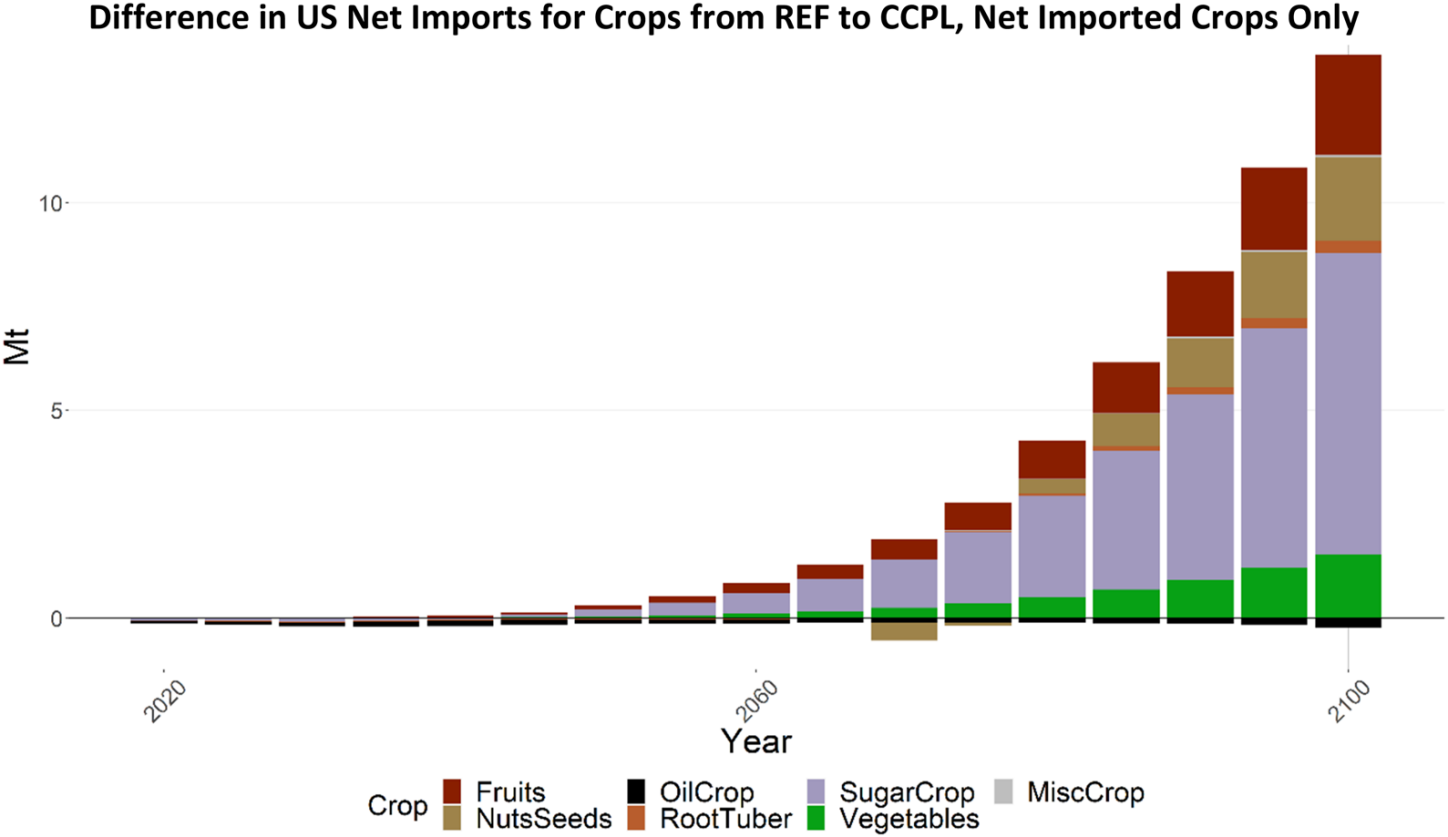
Difference in US net imports for crops. Positive values represent a net increase in imports in CCPL

There are global trade implications associated with incentivizing no-till and cover crop agricultural technologies in the US. The carbon incentives and yield changes from no-till and cover crop practices, along with the changes in crop production they indirectly spur, impact crop prices, which ultimately influence global trade dynamics (Fig. 14). All else equal, the direct effect of valuing carbon in agricultural soil would be to reduce the crop prices as the carbon price incentives are an additional source of revenue to landowners. We see this in all five crops that have no-till and cover crop technologies, where prices are as much as 29% lower in the CCPL case than REF for other grains, and 28% lower for wheat in 2100. For crops that the US imports more of in CCPL (Fig. 15), prices increase.

This crop price reduction could be expected based on the large increase in yields from legume cover cropping for wheat, in addition to the carbon payment. However, these changes also affect the economics of production and trade, leading to increases in production and land planted in wheat at the expense of other crops. The price for soybean, though it also receives a carbon incentive, is only 8% lower in CCPL than in REF. This lower price reduction reflects not just the direct impact of the lower relative increase in SOC and yield for soybean, it also reflects the economic dynamics in the modeling. Although the direct effect of the carbon subsidy on the practices with higher SOC and yields would be to decrease the soybean price, there is a counteracting upward pressure due to the opportunity cost of not growing a crop that has higher SOC potential, like wheat or other grains.

**Fig. 16:**
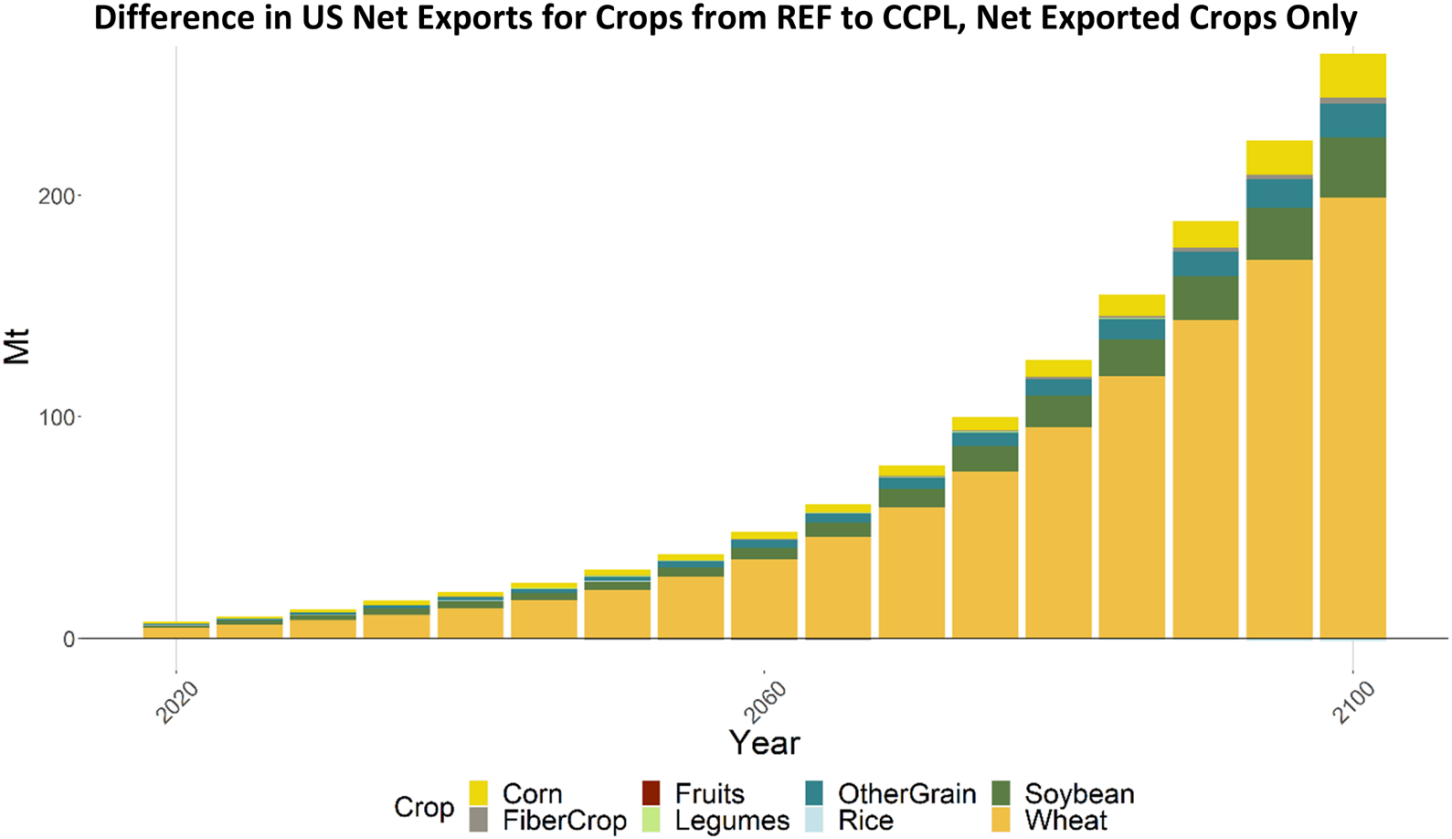
Difference in US net exports for crops. Positive values represent a net increase in exports in CCPL

Comparing REF and CCPL, the US imports substantially more sugar crops (Fig. 15) and exports substantially more wheat (Fig. 16). This is a result of the increased yields and carbon subsidies applied to the higher SOC associated with no-till and cover crop agricultural technologies resulting in increased profitability at decreased prices. Corresponding to the higher yields, the US is able to grow this increased volume without allocating as much additional land as it would without these yield benefits. These changes in import and export volume in the US are counterbalanced by changes in production in the RoW.

## Terrestrial carbon

Carbon dioxide emissions from land-use and land-change are a major consideration in national carbon budgets. To interpret the net impacts of conservation agriculture and the carbon subsidies in CCPL on US and global terrestrial carbon and land use change emissions, we present the land use emissions results in three component steps. First, we show the terrestrial carbon impacts on just US cropland, which can be considered the direct impacts, or first-order, of the scenario. Second, we show the terrestrial carbon impacts on all US land, which also considers any changes outside of cropland. Finally, we show the terrestrial carbon impact on all lands globally; thereby capturing resulting effects of corresponding land use change outside the US.

**Fig. 17:**
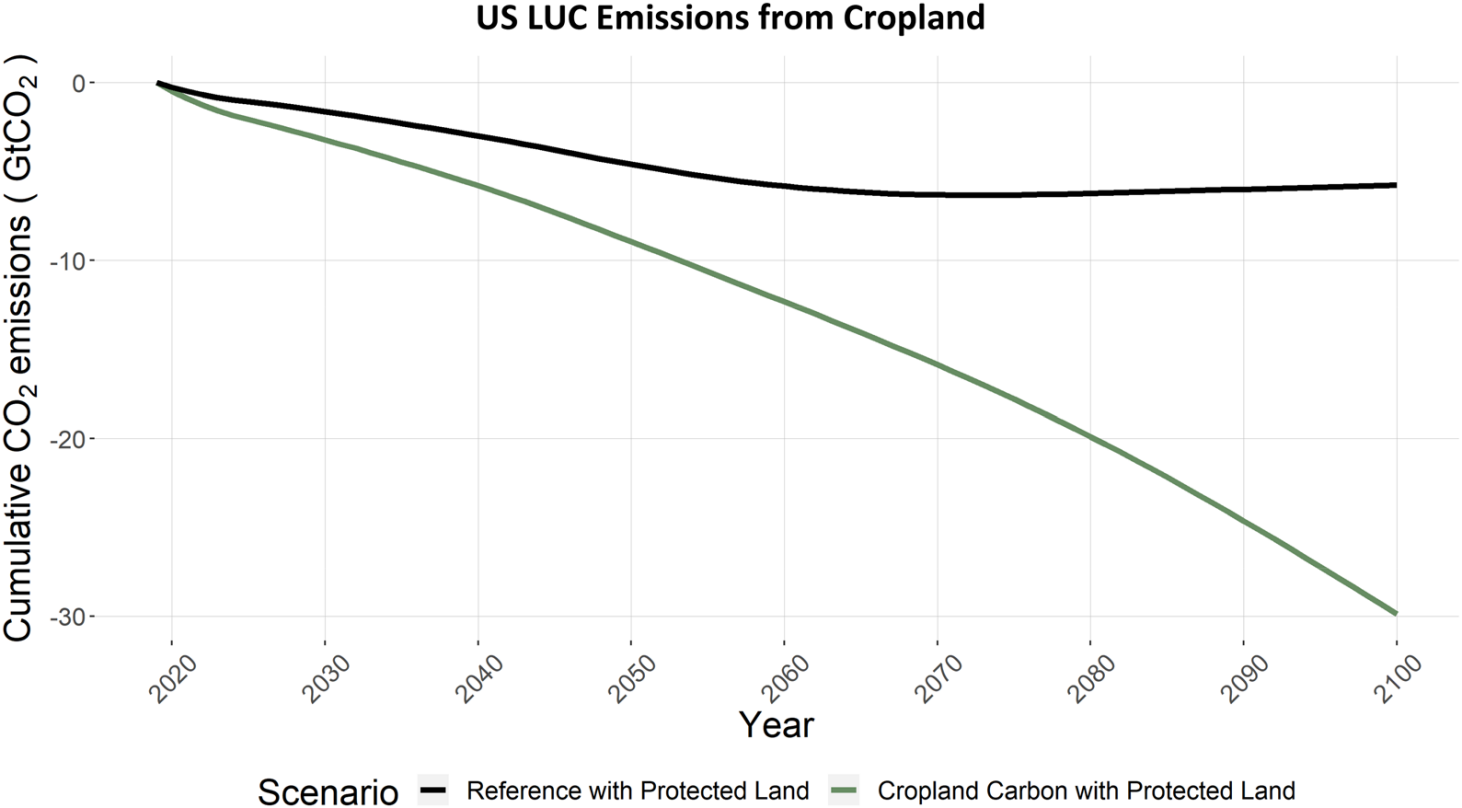
US CO_2_ emissions from land use change in cropland, for REF and CCPL, where negative emissions indicate net terrestrial carbon increases

Figure 17 compares the cumulative terrestrial carbon changes for US cropland in the CCPL and REF scenarios. Results are presented here in terms of cumulative land use change emissions, where a negative result indicates net sequestration or an increase in terrestrial carbon. These results can be interpreted as the change in carbon stored in agricultural soil due to an increase in no-till and cover crop management. From REF to CCPL, net terrestrial carbon in cropland in the US is increased by 24 GtCO_2_ cumulatively to 2100, roughly 0.3 GtCO_2_/year on average. Additionally, no-till and cover crop options only begin ramping up significantly after about 2050 in CCPL (Fig. 5)- there is more potential for carbon uptake if these technologies were adopted sooner. Additional file 1: Figure S1 indicates that a more aggressive carbon price pathway (Carbon_275) results in a higher share of cropland allocated to no-till and cover crop options earlier on. The increased terrestrial carbon sink in the US under CCPL is notable for the global carbon budget (Fig. 19) relative to REF.

**Fig. 18:**
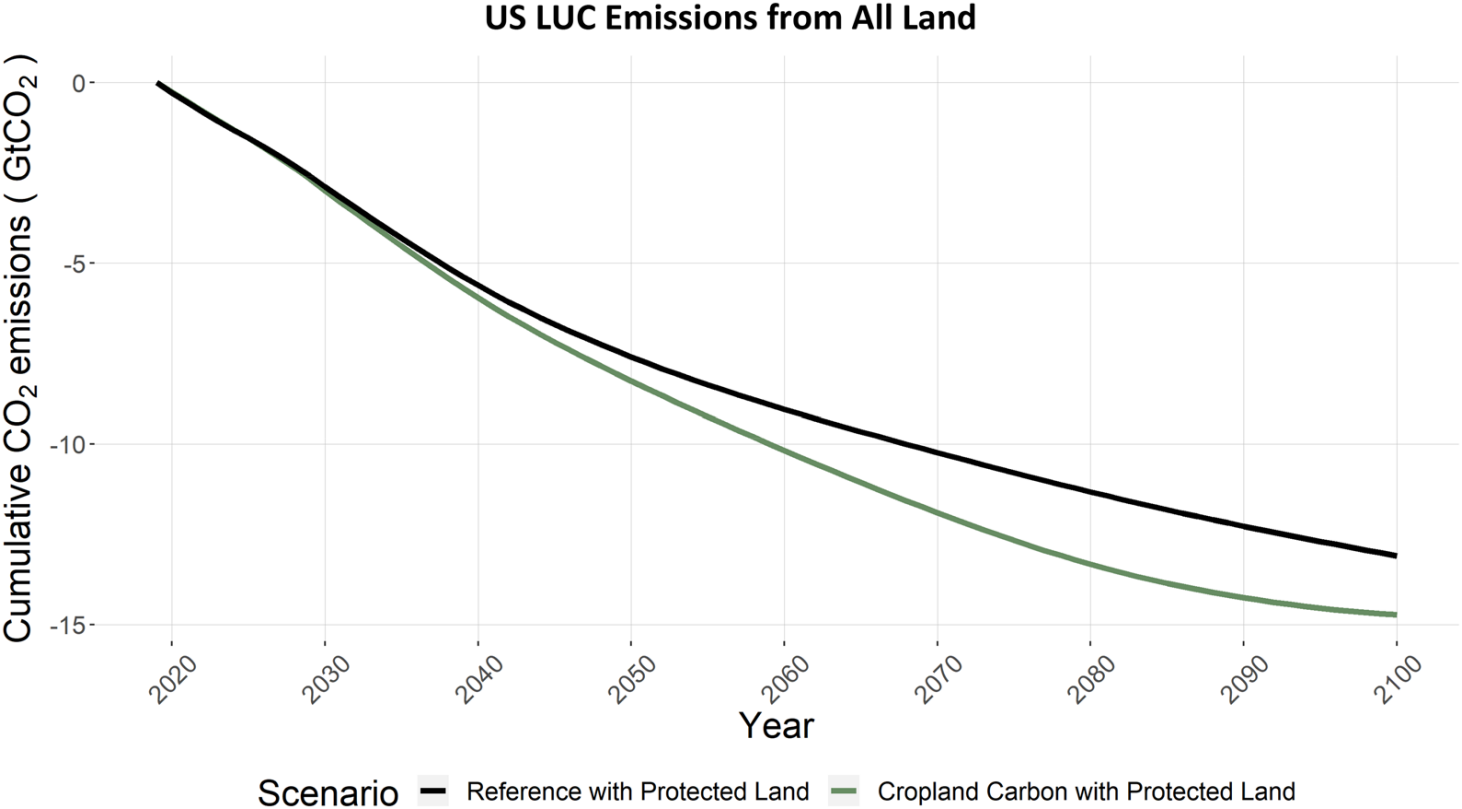
US CO_2_ emissions from land use change, for REF and CCPL, where negative emissions indicate net terrestrial carbon increases

Figure 18 broadens the perspective from Fig. 17 and shows the cumulative changes in terrestrial carbon for all land in the US, including the effects of the cropland expansion induced by CCPL. In the REF scenario as constructed here with a protected land assumption in place, cumulative net CO_2_ emissions from all land use change in the US remain negative, again indicating a net sequestration of carbon into the terrestrial system, reaching -13 GtCO_2_ cumulatively to 2100 (Fig. 18). In contrast to the cropland-only results (Fig. 17), there is just a slight increase in cumulative terrestrial carbon of 2 GtCO_2_ in the CCPL scenario. Recall that one outcome of CCPL was an expansion of US cropland (Fig. 5) due to the increased profitability of crops in this scenario. This increase comes at the expense of other land types in the US – some with higher terrestrial carbon densities. This reduced impact can be attributed to increased LUC emissions from deforestation and reductions in other natural land in the US (Additional file 1: Figure S5) that nearly completely offset the domestic emissions reductions on US cropland. However, the results are still incomplete at this step, and the ultimate result is the net impact on global LUC emissions from this domestic change.

**Fig. 19:**
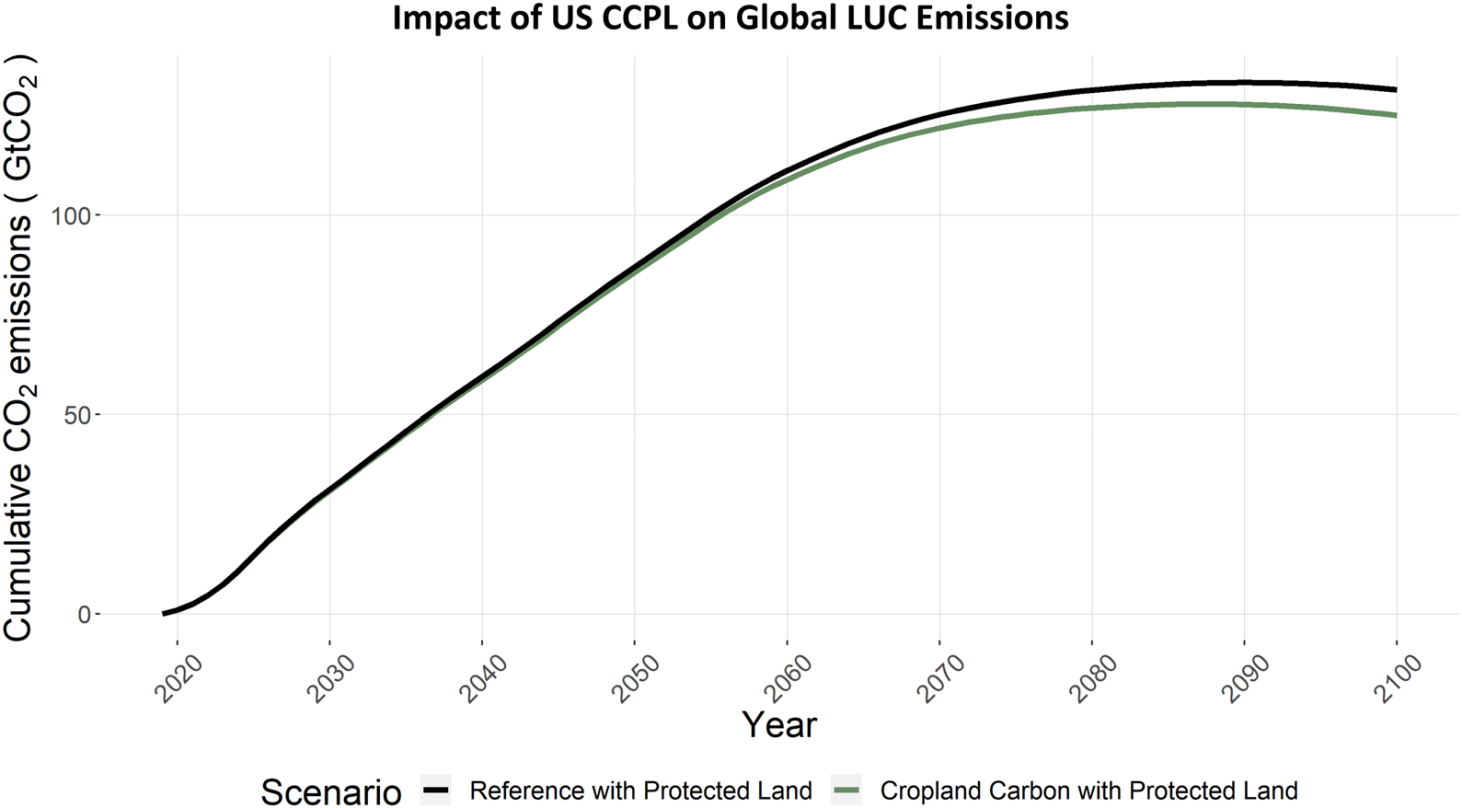
Global CO_2_ emissions from land use change, for REF and CCPL

Figure 19 shows the impact of this US-based policy on global LUC emissions, where emissions are reduced 6.5 GtCO_2_ cumulatively to 2100 in CCPL. This terrestrial carbon impact is larger than the US impact in Fig. 18 because of the increased US crop production and trade with other regions. The increase in cropland in the US to no-till and cover cropping technologies with higher yields lead to higher net crop exports from the US, which allows reduced crop production in other regions globally. The relative reduction in cropland outside the US in CCPL allows a higher amount of natural land, including forests, in CCPL in many regions. For example, in CCPL, Australia and New Zealand can collectively reduce land allocated to wheat by 0.05 million km^2^ (27%) and increase forest, grassland, and pasture (forest change from REF to CCPL in 2100, Additional file 1: Figure S5). While the direct impacts on terrestrial carbon from no-till and cover crops in the US are an important result, these integrated, global, impacts represent the ultimate net impact on terrestrial carbon. This net global impact from US cropland actions in CCPL is about 0.16 GtCO_2_/year on average over 40 years, a conservative but plausible US proportion of the global estimates of 1.4–2.3 GtCO_2_-eq yr^−1^.(3)

## Discussion

With this study, we were able to assess a suite of land use, terrestrial carbon, emissions, and economic impacts of increased no-till and cover crop agriculture in the US in an integrated context that accounts for the global market dynamics. Our results suggest that valuing soil carbon incentivizes no-till and cover cropping, and therefore increases the adoption of these land management practices. Increased use of these practices can in turn have consequential impacts on soil carbon storage and contribute to land-based carbon mitigation. Given the extreme variation we found in the global land-use impacts for different carbon valuation sensitivities, the viability of no-till and cover cropping in the US to make a net global improvement in atmospheric greenhouse gas (GHG) budgets is highly dependent on policy. Furthermore, these results suggest that because of their higher yields and soil carbon sequestration potential over time, no-till and cover crop agricultural technologies are often a more favorable option than their conventional counterparts when there is a carbon price in place.

In addition to showing the impacts of carbon policy on no-till and cover crop agriculture, our sensitivities showed the potential counterintuitive impacts of alternative applications of such policies. For example, by incentivizing no-till land management and addition of cover crops with a carbon price on all terrestrial carbon (AC sensitivity), there is subsequent pressure to increase forested areas and decrease cropland. In addition, by having a carbon price in the US only, there are international implications beyond what only the expansion of these practices (without the addition of a carbon price) in the US would be, because of afforestation and pressure to reduce cropland. Alternatively, when there is a carbon price only on cropland soil carbon (CC sensitivity), cropland expands at the expense of natural land and forests. For these reasons, we added a more targeted policy that only values soil carbon on cropland and protects natural land (CCPL) which allows us to focus on the effects of no-till and cover crop agriculture in the US without significant global forest impacts or unrealistic agricultural expansion. These results could also be interpreted as the impacts of an economic incentive to grow cover crops or reduce tillage, not explicitly implemented by valuing soil carbon, like the mechanism used in this study to promote these practices.

When a technological shift occurs, the economic impacts must be considered. In the case of this study, a change in agricultural practices in the US has global trade implications. Due to different crops having varying yield and SOC benefits from switching to no-till and cover crop agricultural practices, there was more incentive to grow some crops, especially wheat, over others. This resulted in more land allocated to the crops with higher yields and SOC potential, and thus a greater supply of them in the US, and a corresponding decrease in other regions. In general, crops with greater relative SOC increases from no-till and cover cropping saw an increase in production at lower crop prices resulting from the carbon subsidy compared to REF by 2100, while crops with less SOC increase experienced a lower increase in production. In response to a greater production and lower price, the US was able to export significantly more wheat compared to other crops.

To the best of our knowledge, there are no other integrated assessments of the global impacts of policy incentives for adopting no-till or cover cropping within a single country, although several regional studies have been carried out. In lower Manitoba, Canada, shifts towards conservation agriculture were associated with higher quality of life indexes for all citizens [42]. Within France, the adoption of no-till was associated with improved sustainability metrics but had to be paired with crop diversification in order to maintain yields [43]. In crop-livestock systems in Brazil, the adoption of direct seeding, a type of combined cover cropping with no-till, was dependent on the cost of fodder for livestock [44]. Other work has shown that up to 81% of global arable land has the potential to be under conservation agriculture in a broad sense, predominantly to prevent soil erosion [45]. All of these studies emphasized the peripheral benefits of sustainable agricultural practices above and beyond climate change mitigation.

The literature indicates that there is a great deal of uncertainty regarding the long-term physical impacts of no-till and cover crop practices on SOC and yields [4, 5, 7, 10]. By necessity, our incorporation of no-till and cover crop agricultural practices into GCAM involved some simplification of physical characteristics and spatial resolution. However, integrated assessment modeling is a valuable approach for providing an external context for assumptions about physical responses and their potential relative impacts on the larger agriculture, land use, and emissions outcomes. Additional developments to this study could include non-CO_2_ GHG impacts, above ground carbon storage changes, and capital and operating cost considerations. Varying these parameters by technology would further distinguish them and drive more realistic dynamics in GCAM.

Our approach combining biogeochemical modeling (DayCent) of specific management practices in specific regions with an integrated assessment model (GCAM) representing the economics of the agriculture and land use system gives us an internally consistent framework for parameterizing the model and exploring the potential scale and impact of the adoption of these crop practices in the US. This study highlights the long-term land-based carbon mitigation potential from increased conservation agricultural practices, and considers the global land, emissions, and economic impacts. This approach could be extended to model a suite of conservation agricultural technologies in other regions, and potentially be expanded to include additional land-based mitigation strategies falling under the conservation agriculture umbrella such as biochar and alternative grazing.

## Conclusion

When incorporated into GCAM, results under different carbon management incentives indicate that a shift to no-till and cover cropping practices could increase the terrestrial carbon sink with limited effects on crop availability for food and fodder markets. By incentivizing soil carbon, the allocation of land to these management practices increases over time due to their potential to increase SOC. Additionally, these agricultural practices often see increased yields, as well as other co-benefits such improved soil structure, water retention, and nutrient content that further encourage switching from conventional management. While GCAM cannot capture the farm-level intricacies of shifting agricultural practices, this study provides a comprehensive analysis on the potential land, climate, and economic impacts in an integrated, global context.

### Supplementary Information

**Additional file 1. Table S1.** presents a summary of meta-analyses examining the effects that cover crops have on primary crop yield and soil organic carbon (SOC). **Figure S1.** Total cropland allocated to aggregate agricultural technologies in the US, by carbon price sensitivity scenario. Management regimes: C: conventional tillage; N: no-till; F: Fallow; Non-Lgm: Non-legume cover crop; Lgm: Legume cover crop. **Figure S2.** US CO_2_ emissions from land use change for the carbon price sensitivity scenarios. **Figure S3.** Percent change in forest allocation in each of the GCAM regions in 2100 (AC – REF). A positive percent change represents an afforestation response in AC. **Figure S4.** Percent change in forest allocation in each of the GCAM regions in 2100 (CC – REF). A positive percent change represents an afforestation response in CC. **Figure S5.** Percent change in forest allocation in each of the GCAM regions in 2100 (CCPL – REF). A positive percent change represents an afforestation response in CCPL. **Figure S6.** Percent change in yield from conventional-fallow technology for fiber crop and other grain. Error bars represent one standard deviation of the mean of irrigated/rainfed and hi/lo technologies, averages are plotted. **Figure S7.** Percent change in SOC potential from conventional-fallow technology for fiber crop and other grain. Error bars represent one standard deviation of the mean of irrigated/rainfed and hi/lo technologies, averages are plotted. **Figure S8.** Cropland allocation in the US, by crop, for REF and CCPL. “OtherCrop” is an aggregate group of fruits, nuts, seeds, vegetables, purpose-grown biomass, and fodder.

## Data Availability

The datasets supporting the conclusions of this article will be made available at https://github.com/marideeweber/gcam6p0_no_till_CC when the repository is minted.

## Abbreviations

AFOLU: Agriculture, forestry, bioenergy, and other land-use
AC: All Carbon
C_F: Conventional-fallow
C_Lgm: Conventional-legume
C_NonLgm: Conventional-NonLegume
CC: Cropland Carbon
CCPL: Cropland Carbon with Protected Land
DayCent: Daily Century
GCAM: Global Change Analysis Model
IAM: Integrated assessment model
IPCC: Intergovernmental Panel on Climate Change
IRR: Irrigated
N_F: No-till-fallow
N_Lgm: No-till-legume
N_NonLgm: No-till-NonLegume
NRCS: Natural Resources Conservation Service
RFD: Rainfed
REF: Reference with Protected Land
SOC: Soil organic carbon
US: United States
USDA: United States Department of Agriculture

## References

[1] The White House. The White House. 2021 [cited 2022 Sep 26]. Executive Order on Tackling the Climate Crisis at Home and Abroad.https://www.whitehouse.gov/briefing-room/presidential-actions/2021/01/27/executive-order-on-tackling-the-climate-crisis-at-home-and-abroad/. Accessed on 7 Mar 2024

[2] Roe S, Streck C, Obersteiner M, Frank S, Griscom B, Drouet L (2019). Contribution of the land sector to a 1.5 °C world. Nat Clim Chang.

[3] IPCC. Climate Change 2022 Mitigation of Climate Change [Internet]. 2022 [cited 2023 May 12]. https://www.ipcc.ch/report/ar6/wg3/downloads/report/IPCC_AR6_WGIII_Full_Report.pdf. Accessed on 7 Mar 2024

[4] Zomer RJ, Bossio DA, Sommer R, Verchot LV (2017). Global sequestration potential of increased organic carbon in cropland soils. Sci Rep.

[5] Griscom BW, Adams J, Ellis PW, Houghton RA, Lomax G, Miteva DA (2017). Natural climate solutions. Proc Natl Acad Sci USA.

[6] Powlson D, Stirling C, Jat M, Gerard B, Palm C, Sanchez P (2014). Limited potential of no-till agriculture for climate change mitigation. Nat Clim Chang.

[7] Su Y, Gabrielle B, Makowski D (2021). A global dataset for crop production under conventional tillage and no tillage systems. Sci Data.

[8] Manley J, van Kooten GC, Moeltner K, Johnson DW (2005). Creating Carbon offsets in agriculture through no-till cultivation: a meta-analysis of costs and carbon benefits. Clim Change.

[9] Haddaway NR, Hedlund K, Jackson LE, Kätterer T, Lugato E, Thomsen IK (2017). How does tillage intensity affect soil organic carbon? a systematic review. Environmental Evidence.

[10] Nicoloso RS, Rice CW (2021). Intensification of no-till agricultural systems: an opportunity for carbon sequestration. Soil Sci Soc Am J.

[11] Wallander S, Smith D, Bowman M, Claassen R. Cover Crop Trends, Programs, and Practices in the United States. U.S. Department of Agriculture; 2021.

[12] Paustian K, Collier S, Baldock J, Burgess R, Creque J, DeLonge M (2019). Quantifying carbon for agricultural soil management: from the current status toward a global soil information system. Carbon Management.

[13] Moinet GYK, Hijbeek R, van Vuuren DP, Giller KE (2023). Carbon for soils, not soils for carbon. Glob Change Biol.

[14] Powlson DS, Galdos MV (2023). Challenging claimed benefits of soil carbon sequestration for mitigating climate change and increasing crop yields: Heresy or sober realism?. Glob Change Biol.

[15] Lal R, Griffin M, Apt J, Lave L, Morgan MG (2004). Managing Soil Carbon. Science.

[16] Conservation Stewardship Program | Natural Resources Conservation Service [Internet]. [cited 2023 Oct 30]. https://www.nrcs.usda.gov/programs-initiatives/csp-conservation-stewardship-program. Accessed on 7 Mar 2024

[17] Environmental Quality Incentives Program | Natural Resources Conservation Service [Internet]. 2023 [cited 2023 Oct 30]. https://www.nrcs.usda.gov/programs-initiatives/eqip-environmental-quality-incentives. Accessed on 7 Mar 2024

[18] USDA Offers Expanded Conservation Program Opportunities to Support Climate Smart Agriculture in 2022 [Internet]. [cited 2023 Oct 30]. https://www.usda.gov/media/press-releases/2022/01/10/usda-offers-expanded-conservation-program-opportunities-support. Accessed on 7 Mar 2024

[19] Luo Z, Wang E, Sun OJ (2010). Can no-tillage stimulate carbon sequestration in agricultural soils? A meta-analysis of paired experiments. Agr Ecosyst Environ.

[20] Ogle SM, Alsaker C, Baldock J, Bernoux M, Breidt FJ, McConkey B (2019). Climate and soil characteristics determine where no-till management can store carbon in soils and mitigate greenhouse gas emissions. Sci Rep.

[21] Abdalla M, Hastings A, Cheng K, Yue Q, Chadwick D, Espenberg M (2019). A critical review of the impacts of cover crops on nitrogen leaching, net greenhouse gas balance and crop productivity. Glob Chang Biol.

[22] Marcillo G, Miguez F (2017). Corn yield response to winter cover crops: an updated meta-analysis. J Soil Water Conserv.

[23] Garba II, Bell LW, Williams A (2022). Cover crop legacy impacts on soil water and nitrogen dynamics, and on subsequent crop yields in drylands: a meta-analysis. Agron Sustain Dev.

[24] Zhao X, Liu SL, Pu C, Zhang XQ, Xue JF, Ren YX (2017). Crop yields under no-till farming in China: a meta-analysis. Eur J Agron.

[25] Pittelkow CM, Linquist BA, Lundy ME, Liang X, van Groenigen KJ, Lee J (2015). When does no-till yield more?. A global meta-analysis Field Crops Research.

[26] Huang Y, Ren W, Wang L, Hui D, Grove JH, Yang X (2018). Greenhouse gas emissions and crop yield in no-tillage systems: a meta-analysis. Agr Ecosyst Environ.

[27] Del Grosso SJ, Parton WJ, Mosier AR, Hartman M, Brenner J, Ojima D, et al. Simulated interaction of carbon dynamics and nitrogen trace gas fluxes using the DAYCENT model. In: Modeling carbon and nitrogen dynamics for soil management. 2001. p. 303–32.

[28] Parton WJ, Hartman M, Ojima D, Schimel D (1998). DAYCENT and its land surface submodel: description and testing. Global Planet Change.

[29] National Agricultural Statistics Service, United States Department of Agriculture. 2017 Census of Agriculture [Internet]. 2019. Report No: Volume 1. https://www.nass.usda.gov/Publications/AgCensus/2017/Full_Report/Volume_1,_Chapter_1_US/usv1.pdf. Accessed on 7 Mar 2024

[30] Thornton MM, Shrestha R, Wei Y, Thornton PE, Kao SC, Wilson BE. Daymet: Daily Surface Weather Data on a 1-km Grid for North America, Version 4 R1. ORNL DAAC [Internet]. 2022 Nov 1 [cited 2023 Oct 24]; https://daac.ornl.gov/cgi-bin/dsviewer.pl?ds_id=2129. Accessed on 7 Mar 2024

[31] Calvin K, Patel P, Clarke L, Asrar G, Bond-Lamberty B, Cui RY (2019). GCAM v51: representing the linkages between energy, water, land, climate, and economic systems. Geosci Model Dev.

[32] Edmonds J, Reilly J (1986). Global energy: assessing the future. Energy Policy.

[33] Calvin K, Wise M, Kyle P, Patel P, Clarke L, Edmonds J (2013). Trade-offs of different land and bioenergy policies on the path to achieving climate targets. Clim Change.

[34] Wise M, Calvin K, Thomson A, Clarke L, Bond-Lamberty B, Sands R (2009). Implications of limiting CO2 concentrations for land use and energy. Science.

[35] Wise M, Calvin K, Kyle P, Luckow P, Edmonds J (2014). Economic and physical modeling of land use in GCAM 3.0 and an application to agricultural productivity, land, and terrestrial carbon. Clim Change Econ.

[36] Wise M, Hodson E, Mignone B, Clarke L, Waldhoff S, Luckow P (2015). An approach to computing marginal land use change carbon intensities for bioenergy in policy applications - ScienceDirect. Energy Economics.

[37] Snyder A, Calvin K, Clarke L, Edmonds J, Kyle P, Narayan K (2020). The domestic and international implications of future climate for U.S. agriculture in GCAM. PLoS ONE.

[38] Wise M, Dooley J, Luckow P, Calvin K, Kyle P (2014). Agriculture, land use, energy and carbon emission impacts of global biofuel mandates to mid-century. Appl Energ.

[39] Wise M, McJeon H, Calvin K, Clarke L, Kyle P (2014). Assessing the Interactions among U.S. climate policy, biomass energy, and agricultural trade. Energy J.

[40] Bond-Lamberty B, Dorheim K, Cui R, Horowitz R, Snyder A, Calvin K (2019). gcamdata: an r package for preparation, synthesis, and tracking of input data for the GCAM integrated human-earth systems model. J Open Res Software.

[41] Rennert K, Errickson F, Prest BC, Rennels L, Newell RG, Pizer W (2022). Comprehensive evidence implies a higher social cost of CO2. Nature.

[42] Sharma T, Carmichael J, Klinkenberg B (2006). Integrated modeling for exploring sustainable agriculture futures. Futures.

[43] Craheix D, Angevin F, Doré T, de Tourdonnet S (2016). Using a multicriteria assessment model to evaluate the sustainability of conservation agriculture at the cropping system level in France. Eur J Agron.

[44] Alary V, Corbeels M, Affholder F, Alvarez S, Soria A, Valadares Xavier JH (2016). Economic assessment of conservation agriculture options in mixed crop-livestock systems in Brazil using farm modelling. Agric Syst.

[45] Prestele R, Hirsch AL, Davin EL, Seneviratne SI, Verburg PH (2018). A spatially explicit representation of conservation agriculture for application in global change studies. Glob Change Biol.

