# Supplementary material for: Potential long-term, global effects of enhancing the domestic terrestrial carbon sink in the United States through no-till and cover cropping
Source: Carbon Balance Manag. 2024 Jun 14;19:18. doi: 10.1186/s13021-024-00256-2 (PMC11179271; doi:10.1186/s13021-024-00256-2)
Supplement: Supplementary file 1 — Additional file 1. Table S1. presents a summary of meta-analyses examining the effects that cover crops have on primary crop yield and soil organic carbon (SOC). Figure S1. Total cropland allocated to aggregate agricultural technologies in the US, by carbon price sensitivity scenario. Management regimes: C: conventional tillage; N: no-till; F: Fallow; Non-Lgm: Non-legume cover crop; Lgm: Legume cover crop. Figure S2. US CO2 emissions from land use change for the carbon price sensitivity scenarios. Figure S3. Percent change in forest allocation in each of the GCAM regions in 2100 (AC – REF). A positive percent change represents an afforestation response in AC. Figure S4. Percent change in forest allocation in each of the GCAM regions in 2100 (CC – REF). A positive percent change represents an afforestation response in CC. Figure S5. Percent change in forest allocation in each of the GCAM regions in 2100 (CCPL – REF). A positive percent change represents an afforestation response in CCPL. Figure S6. Percent change in yield from conventional-fallow technology for fiber crop and other grain. Error bars represent one standard deviation of the mean of irrigated/rainfed and hi/lo technologies, averages are plotted. Figure S7. Percent change in SOC potential from conventional-fallow technology for fiber crop and other grain. Error bars represent one standard deviation of the mean of irrigated/rainfed and hi/lo technologies, averages are plotted. Figure S8. Cropland allocation in the US, by crop, for REF and CCPL. “OtherCrop” is an aggregate group of fruits, nuts, seeds, vegetables, purpose-grown biomass, and fodder. [file 13021_2024_256_MOESM1_ESM.docx]

**Potential long-term, global effects of enhancing the domestic terrestrial carbon sink in the United States through no-till and cover cropping**

**Maridee Weber^1*^, Marshall Wise^1^, Patrick Lamers^2^, Yong Wang^2^, Greg Avery^2^, Kendalynn A. Morris^1^, Jae Edmonds^1^**

^1^ *Joint Global Change Research Institute (Pacific Northwest National Laboratory and University of Maryland), 5825 University Research Court, College Park, MD, USA*

*^2^ National Renewable Energy Laboratory, 15013 Denver W Pkwy, Golden, CO, USA*

**Corresponding author:* [*maridee.weber@pnnl.gov*](mailto:maridee.weber@pnnl.gov)

# Supplementary Materials

Table S1 presents a summary of meta-analyses examining the effects that cover crops have on primary crop yield and soil organic carbon (SOC).

| **Meta-analyses on cover crops** | | | | **Yield** | | | **SOC** | | |
| --- | --- | --- | --- | --- | --- | --- | --- | --- | --- |
| **Study** | **Year** | **Crop** | **Region** | **Legume** | **Non-Legume** | **Mixed** | **Legume** | **Non-Legume** | **Mixed** |
| Abdalla et al. (1) | 2019 | All | Global | ↓ | ↓ | ↑ | ↑ | ↑ | none/ minor ↓ |
| Poeplau & Don (2) | 2015 | All | Global |  |  |  | ↑ | ↑ | ↑ |
| Young et al. (3) | 2021 | All | Global |  |  | ↑ |  |  | ↑ |
| McClelland et al. (4) | 2020 | All | Global |  |  |  | ↑ | ↑ | ↑ |
| Jian et al. (5) | 2020 | All | Global |  |  |  | ↑ | ↑ | ↑ |
| Marcilla & Miguez (6) | 2017 | Corn | US & Canada | ↑ | none/minor | ↑ |  |  |  |
| Garba et al. (7) | 2022 | All | Global-arid climates | ↓ | ↓ | ↓ |  |  |  |

Table S1: Effects of cover crops on primary crop yield and SOC from literature.

**Appendix I: Sensitivities**

**Sensitivity: Carbon Price**

Five carbon price sensitivity scenarios were developed to assess the impacts of different carbon prices on key metrics, such as land allocation (Figure S1) and land use change emissions (Figure S2). Each carbon price scenario is named based on its starting carbon price in 2020, and all prices increase at a rate of 5% annually. Additionally, all scenarios assume 90% of previously undeveloped lands are protected from expansion of managed land-use in the US. For example, Carbon_183 represents our CCPL scenario, with a carbon price of $183.50 in 2020.

Figure S1 shows total cropland allocation in the US by tillage and cover crop. Higher starting carbon prices, like in Carbon_92, Carbon_183, and Carbon_275 result in a greater increase of cropland allocated to no-till technologies by the end of the century, while lower or no carbon prices (Carbon_0, Carbon_18) see little change in technology shares from 2020 to 2100. Because we were interested in assessing the impacts of an increase in no-till and cover crop technologies, the Carbon_0 and Carbon_18 price pathways did not make sense to use.

**US Cropland Allocation by Tillage and Cover Crop**


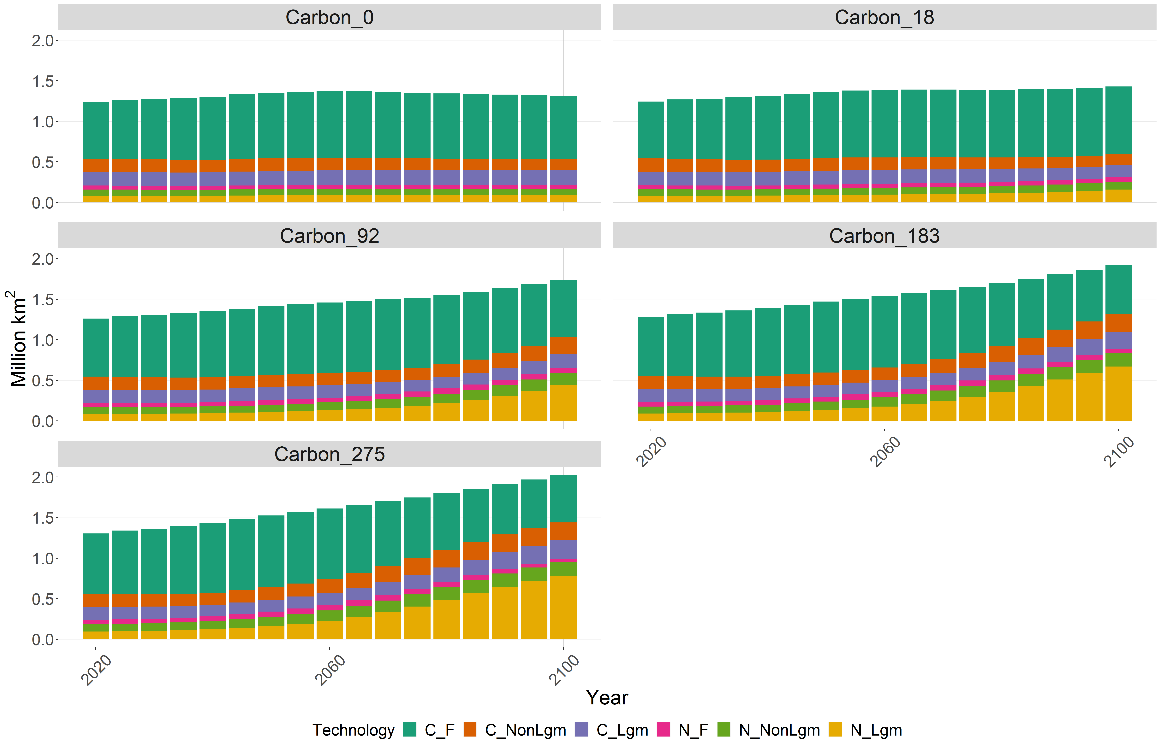


Figure S1: Total cropland allocated to aggregate agricultural technologies in the US, by carbon price sensitivity scenario. Management regimes: C: conventional tillage; N: no-till; F: Fallow; Non-Lgm: Non-legume cover crop; Lgm: Legume cover crop

Figure S2 shows cumulative CO_2_ emissions from land use change in the US for our five carbon price sensitivity scenarios. By 2100, cumulative emissions are similar among our three remaining pathways, with -14.86 GtCO_2_/year in Carbon_92, -14.72 GtCO_2_/year in Carbon_183, and -14.46 GtCO_2_/year in Carbon_275. Because of this similarity, we ultimately selected Carbon_183 as our carbon price pathway for our CCPL scenario. This selection is additionally supported by a recent study that estimates the social cost of carbon is $183.50 (8).

**US LUC Emissions**


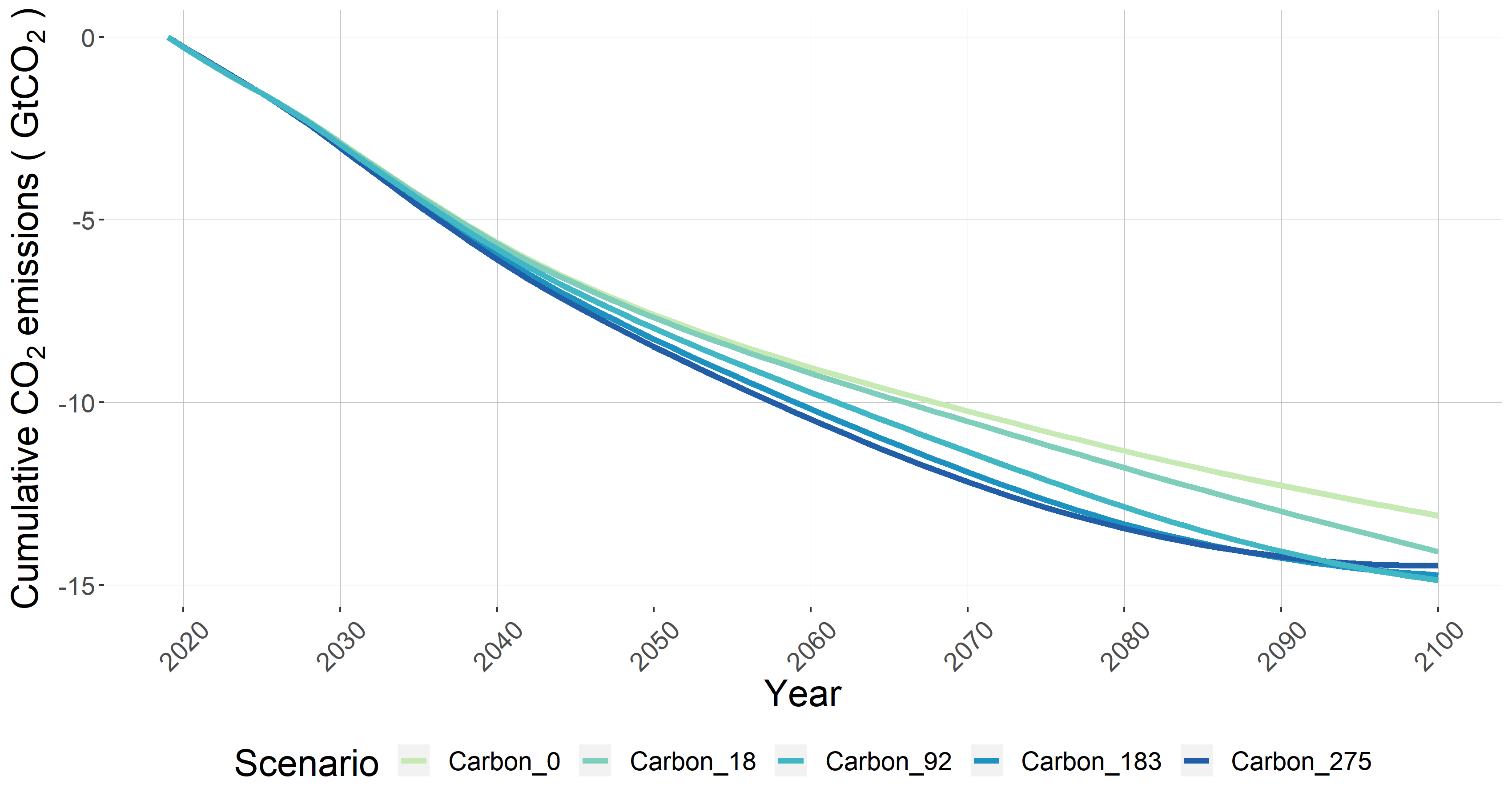


Figure S2: US CO_2_ emissions from land use change for the carbon price sensitivity scenarios.

**Sensitivity: Policy Target**

Figure 5 in the main text shows US cropland responses to different carbon pricing mechanisms in our two scenarios and sensitivities. When all carbon on all land types is valued, including on forested land, such as in the All Carbon (AC) sensitivity, the incentive results in a decrease in total cropland in the US (Figure 5). The decreasing cropland behavior, which is potentially counterintuitive if considered in isolation, can be explained through an afforestation response- because forested land has greater carbon storage potential than cropland and all land carbon is valued, land previously allocated to cropland is replaced by forests, increasing the terrestrial sink. The opposite effect is shown in the Cropland Carbon (CC) sensitivity, where forests and other non-cropland carbon is not valued while agricultural soil carbon is. This results in a deforestation response in the US and an expansion of cropland that is double that of the reference scenario by 2100 (Figure 5). Milder afforestation and deforestation responses are seen in all other regions globally (Figures S3 and S4).

The significant afforestation and deforestation responses in the AC and CC sensitivity scenarios are logical outcomes of these incentives as applied which must be taken into consideration, but they tend to obscure the impact of a policy intended to affect cropland. As a result, we constructed the Reference with Protected Land (REF) and Cropland Carbon with Protected Land (CCPL) scenarios to stabilize total cropland somewhat by removing 90% of natural lands (forest, grassland, etc.) from economic opportunities for commercial land expansion in the US. The 90% “land protection” assumption is meant to be illustrative and should not be considered a reflection of a specific policy. Our intent is to assess the direct impact of terrestrial carbon banking on agriculture in isolation, although the multisector impacts of scenario design are critical. Also, the 90% protection assumption allows for pressures for cropland expansion that arise from the carbon subsidy to be seen in our results, which would not be the case if we assumed 100% protection.

Figures S3, S4, and S5 show the percent change in forested land in each GCAM region, from REF, for AC, CC, and CCPL, respectively. In AC (Figure S3), we see a 19% increase in forested land in the US, and a change of between 0.07% and -4% in all other regions. This increase in US forests is the result of how the sensitivity was designed, with a valuation of all carbon on all lands incentivizing the expansion of forests (carbon dense) more than cropland. In CC (Figure S4), we see a 23% decrease in forested land in the US, and a change of between -0.3% and 6% in all other regions. This decrease in US forests is the result of only valuing agricultural soil carbon, which promotes the expansion of cropland into forested land. In CCPL (Figure S5), we see a less significant reduction of forested land in the US (6%), and a change of between -0.5% and 3.5% in all other regions. The reduction in forested land of only 6% compared to 23% in the CC sensitivity is due to removing 90% of natural lands from economic opportunities for commercial land expansion in the US in CCPL, which restricts the substantial expansion of cropland into forest that was observed in CC.

**Change in Regional Forest Allocation from REF to AC (2100)**


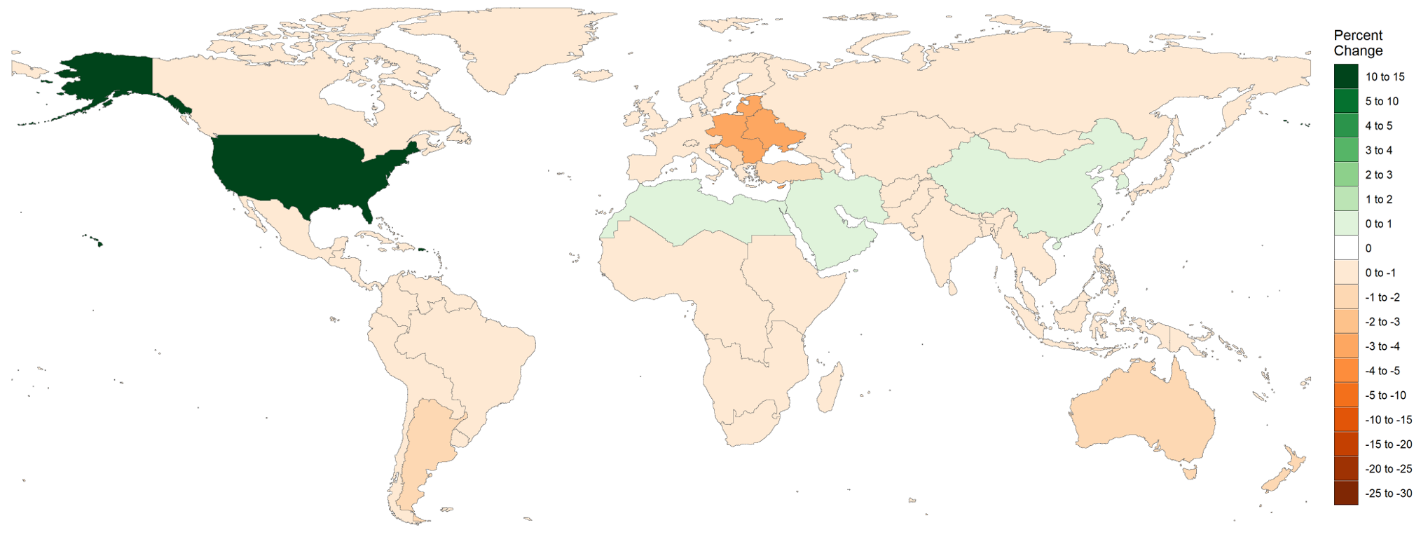


Figure S3: Percent change in forest allocation in each of the GCAM regions in 2100 (AC – REF). A positive percent change represents an afforestation response in AC.

**Change in Regional Forest Allocation from REF to CC (2100)**


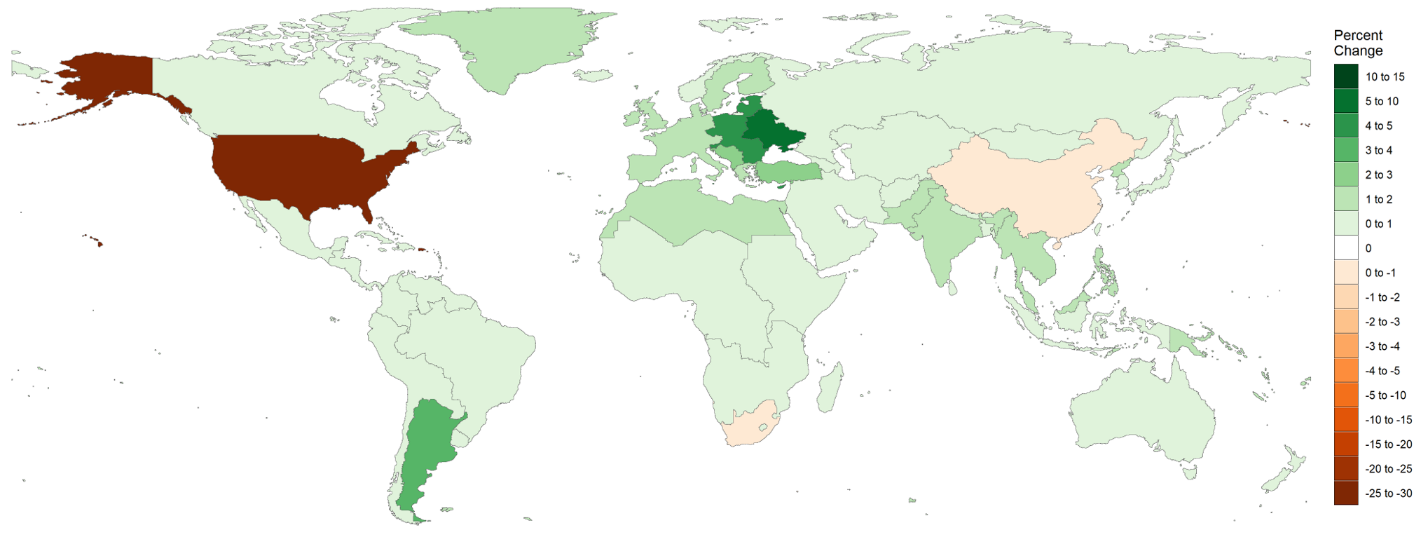


Figure S4: Percent change in forest allocation in each of the GCAM regions in 2100 (CC – REF). A positive percent change represents an afforestation response in CC.

**Change in Regional Forest Allocation from REF to CCPL (2100)**


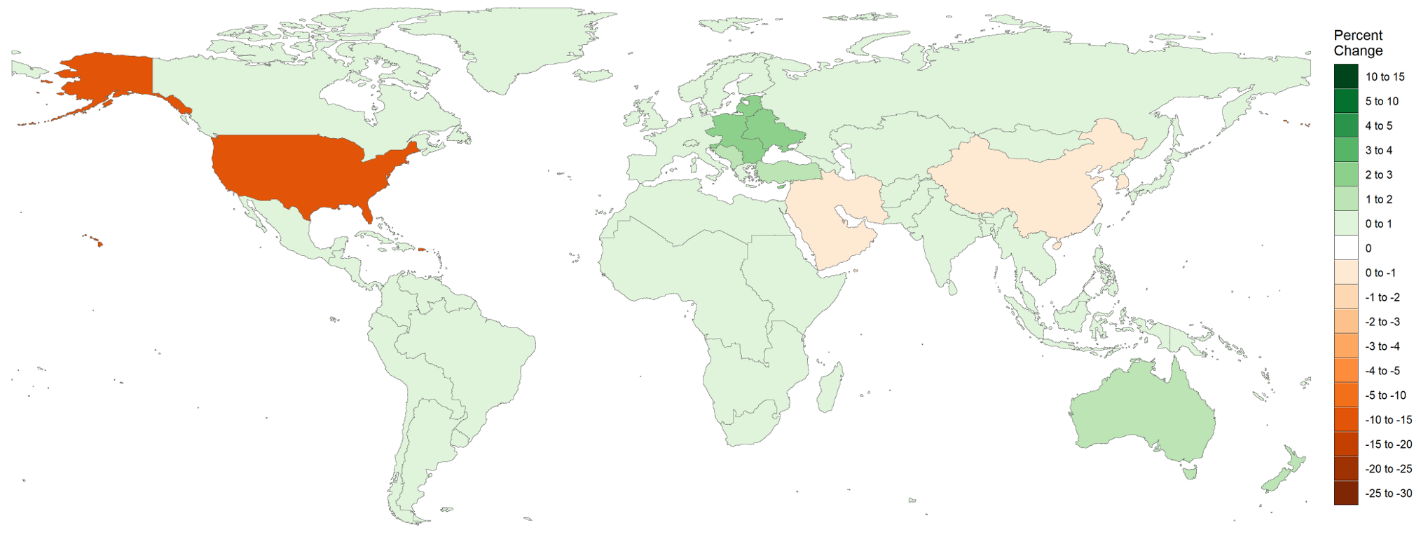


Figure S5 : Percent change in forest allocation in each of the GCAM regions in 2100 (CCPL – REF). A positive percent change represents an afforestation response in CCPL.

**Appendix II: Crop Yield**

Figures S6 and S7 provide yield and SOC change potential for fiber crop and other grain crop categories when switching from conventional-fallow. In Figure S6, both crop groups exhibit higher yields when switching from conventional practices to no-till and cover cropping. Fiber crops with no-till legume regimens can offer especially impressive increases in yield- around 60%. Other grains see yield improvements more similar to corn (Figure 8), with the maximum yield improvement at around 20%. Figure S7 offers details on SOC potential for the same two crop groups. Both have the highest SOC change potential in no-till legume regimens, aside from some other grain conventional non-legume technology options that can reach over 60% change depending on the irrigation/rainfed and fertilizer combination.

**Yield Change from Conventional-Fallow**


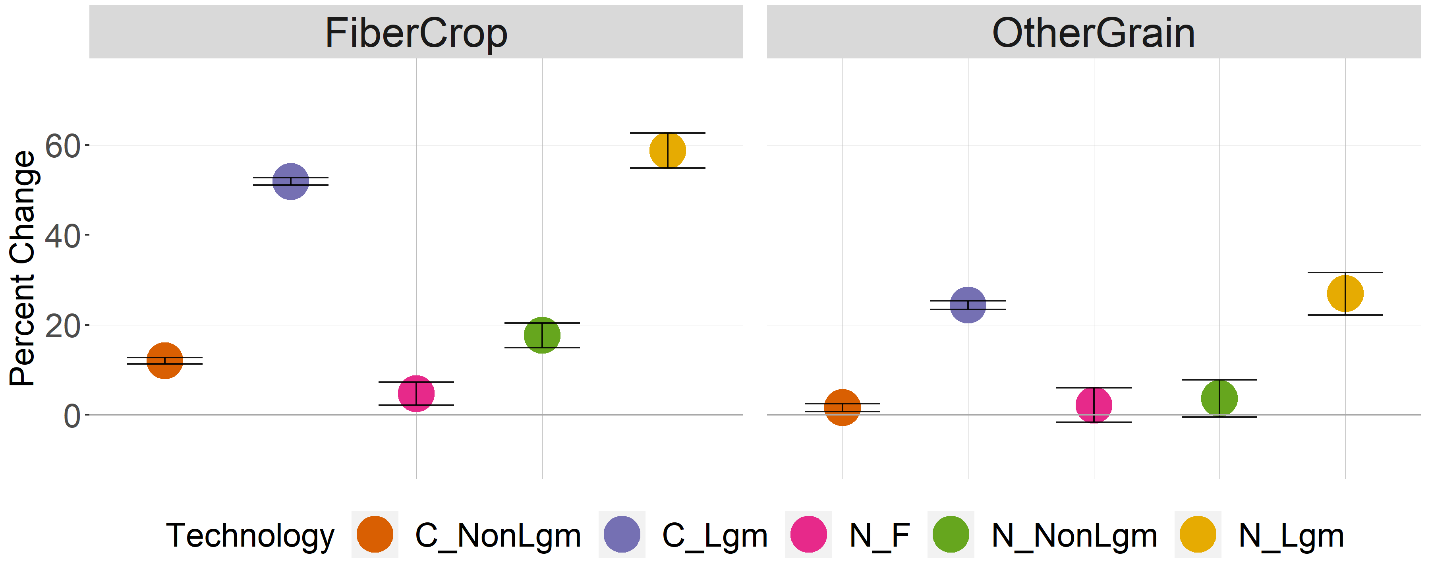


Figure S6: Percent change in yield from conventional-fallow technology for fiber crop and other grain. Error bars represent one standard deviation of the mean of irrigated/rainfed and hi/lo technologies, averages are plotted.

**Change in SOC Potential from Conventional-Fallow**


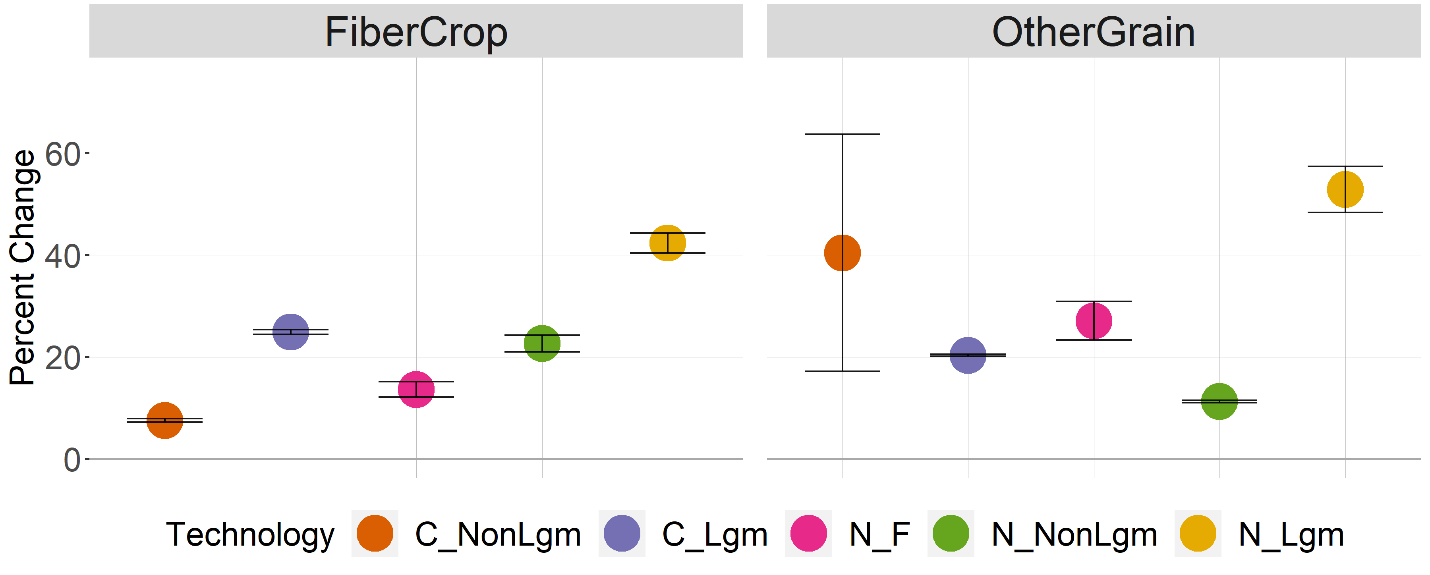


Figure S7: Percent change in SOC potential from conventional-fallow technology for fiber crop and other grain. Error bars represent one standard deviation of the mean of irrigated/rainfed and hi/lo technologies, averages are plotted.

Figure S8 shows total US cropland allocation, by crop, for REF and CCPL. In REF in 2100, total US cropland allocation is 1.3 million km^2^. Besides “OtherCrop”, which is an aggregate group of fruits, nuts, seeds, vegetables, purpose-grown biomass, and fodder, land allocated to corn is 25% of total, soybean is 23%, and wheat is 16%. In CCPL in 2100, total US cropland allocation increases 46% to 1.9 million km^2^. Crop shares also change with 30% of cropland allocated to wheat, 21% to soybean, 20% to corn, and 19% to the aggregate other crop category.

**US Cropland Allocation**


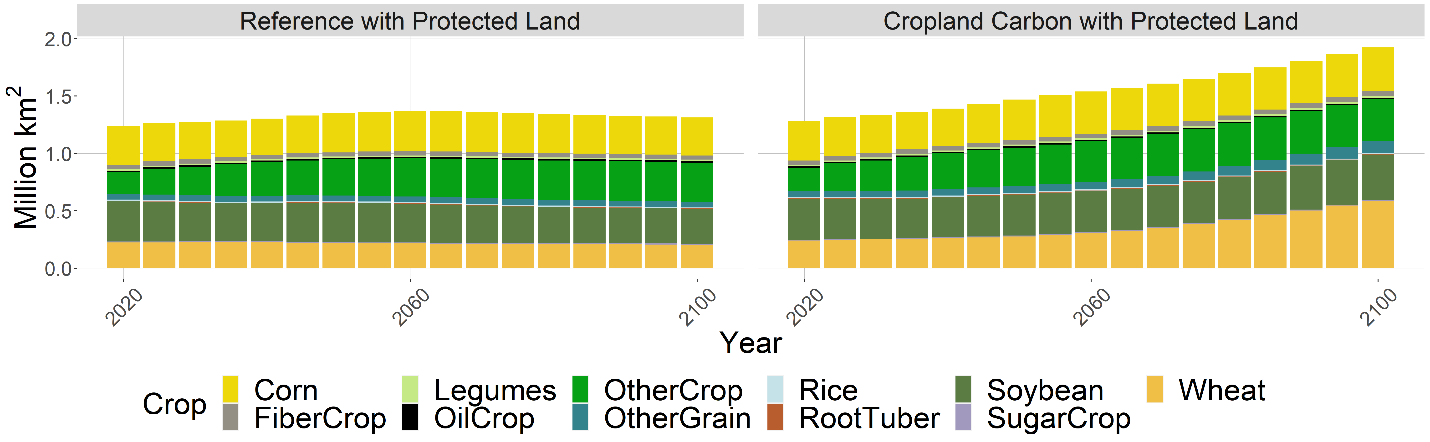


Figure S8: Cropland allocation in the US, by crop, for REF and CCPL. “OtherCrop” is an aggregate group of fruits, nuts, seeds, vegetables, purpose-grown biomass, and fodder.

# References

1. Abdalla M, Hastings A, Cheng K, Yue Q, Chadwick D, Espenberg M, et al. A critical review of the impacts of cover crops on nitrogen leaching, net greenhouse gas balance and crop productivity. Glob Chang Biol. 2019 Aug;25(8):2530–43.

2. Poeplau C, Don A. Carbon sequestration in agricultural soils via cultivation of cover crops – A meta-analysis. Agriculture, Ecosystems & Environment. 2015 Feb 1;200:33–41.

3. Young MD, Ros GH, de Vries W. Impacts of agronomic measures on crop, soil, and environmental indicators: A review and synthesis of meta-analysis. Agriculture, Ecosystems & Environment. 2021 Oct 1;319:107551.

4. McClelland SC, Paustian K, Schipanski ME. Management of cover crops in temperate climates influences soil organic carbon stocks: a meta-analysis. Ecological Applications. 2021;31(3):e02278.

5. Jian J, Du X, Reiter MS, Stewart RD. A meta-analysis of global cropland soil carbon changes due to cover cropping. Soil Biology and Biochemistry. 2020 Apr 1;143:107735.

6. Marcillo G, Miguez F. Corn yield response to winter cover crops: An updated meta-analysis. Journal of Soil and Water Conservation. 2017 May 1;72:226–39.

7. Garba II, Bell LW, Williams A. Cover crop legacy impacts on soil water and nitrogen dynamics, and on subsequent crop yields in drylands: a meta-analysis. Agron Sustain Dev. 2022 Apr 20;42(3):34.

8. Rennert K, Errickson F, Prest BC, Rennels L, Newell RG, Pizer W, et al. Comprehensive evidence implies a higher social cost of CO2. Nature. 2022 Oct;610(7933):687–92.
